# Balancing Extrasynaptic Excitation and Synaptic Inhibition within Olfactory Bulb Glomeruli

**DOI:** 10.1523/ENEURO.0247-19.2019

**Published:** 2019-08-07

**Authors:** David H. Gire, Joseph D. Zak, Jennifer N. Bourne, Noah B. Goodson, Nathan E. Schoppa

**Affiliations:** 1 Department of Physiology and Biophysics, University of Colorado School of Medicine, Aurora, CO 80045; 2 Neuroscience Graduate Program, University of Colorado School of Medicine, Aurora, CO 80045

**Keywords:** excitation inhibition balance, GABA, glutamate, mitral cell, olfactory bulb, transporter

## Abstract

Glutamatergic transmission in the brain typically occurs at well-defined synaptic connections, but increasing evidence indicates that neural excitation can also occur through activation of “extrasynaptic” glutamate receptors. Here, we investigated the underlying mechanisms and functional properties of extrasynaptic signals that are part of a feedforward path of information flow in the olfactory bulb. This pathway involves glutamatergic interneurons, external tufted cells (eTCs), that are excited by olfactory sensory neurons (OSNs) and in turn excite output mitral cells (MCs) extrasynaptically. Using pair-cell and triple-cell recordings in rat bulb slices (of either sex), combined with ultrastructural approaches, we first present evidence that eTC-to-MC signaling results from “spillover” of glutamate released at eTC synapses onto GABAergic periglomerular (PG) cells in glomeruli. Thus, feedforward excitation is an indirect result of and must cooccur with activation of inhibitory circuitry. Next, to examine the dynamics of the competing signals, we assayed the relationship between the number of spikes in eTCs and excitation of MCs or PG cells in pair-cell recordings. This showed that extrasynaptic excitation in MCs is very weak due to single spikes but rises sharply and supralinearly with increasing spikes, differing from sublinear behavior for synaptic excitation of PG cells. Similar dynamics leading to a preference for extrasynaptic excitation were also observed during recordings of extrasynaptic and inhibitory currents in response to OSN input of increasing magnitude. The observed alterations in the balance between extrasynaptic excitation and inhibition in glomeruli with stimulus strength could underlie an intraglomerular mechanism for olfactory contrast enhancement.

## Significance Statement

Glutamatergic transmission in the brain occurs primarily at anatomically defined synaptic connections, but increasing evidence supports the prevalence of “extrasynaptic” mechanisms. Here, we investigated extrasynaptic glutamatergic transmission between two types of excitatory cells in glomeruli of the rodent olfactory bulb, as well as its relationship with respect to local GABAergic inhibition. Our results indicate that weak stimuli preferentially favor inhibition over extrasynaptic excitation, but differences in the non-linear properties between extrasynaptic excitation and inhibition result in strong stimuli favoring extrasynaptic excitation. The shift in balance between extrasynaptic excitation and inhibition with stimulus strength could provide a novel intraglomerular mechanism for olfactory contrast enhancement, helping the brain discriminate different but similar odors.

## Introduction

Glutamatergic transmission in the CNS occurs primarily at anatomically defined synapses, where a presynaptic axon directly apposes a postsynaptic specialization with a high density of glutamate receptors. There are however numerous exceptions to this rule, where glutamate can excite “extrasynaptic” receptors at more distant sites (Asztely et al., 1997; Kullmann and Asztely, 1998). This extrasynaptic transmission, sometimes referred to as “spillover,” may have a number of functions, including driving synaptic plasticity (Scanziani et al., 1996), amplifying coexisting synaptic transmission (DiGregorio et al., 2002; Sargent et al., 2005; Chalifoux and Carter, 2011), or, in some cases, being the primary mechanism of information flow between neurons that lack synaptic connections (Isaacson, 1999; Szapiro and Barbour, 2007; Szmajda and DeVries, 2011; Coddington et al., 2013; Nietz et al., 2017). Extrasynaptic glutamatergic transmission is particularly noteworthy as it could be overlooked by many anatomic methods now being used to map the CNS (Luo et al., 2008; Swanson and Lichtman, 2016).

**Figure 1.**
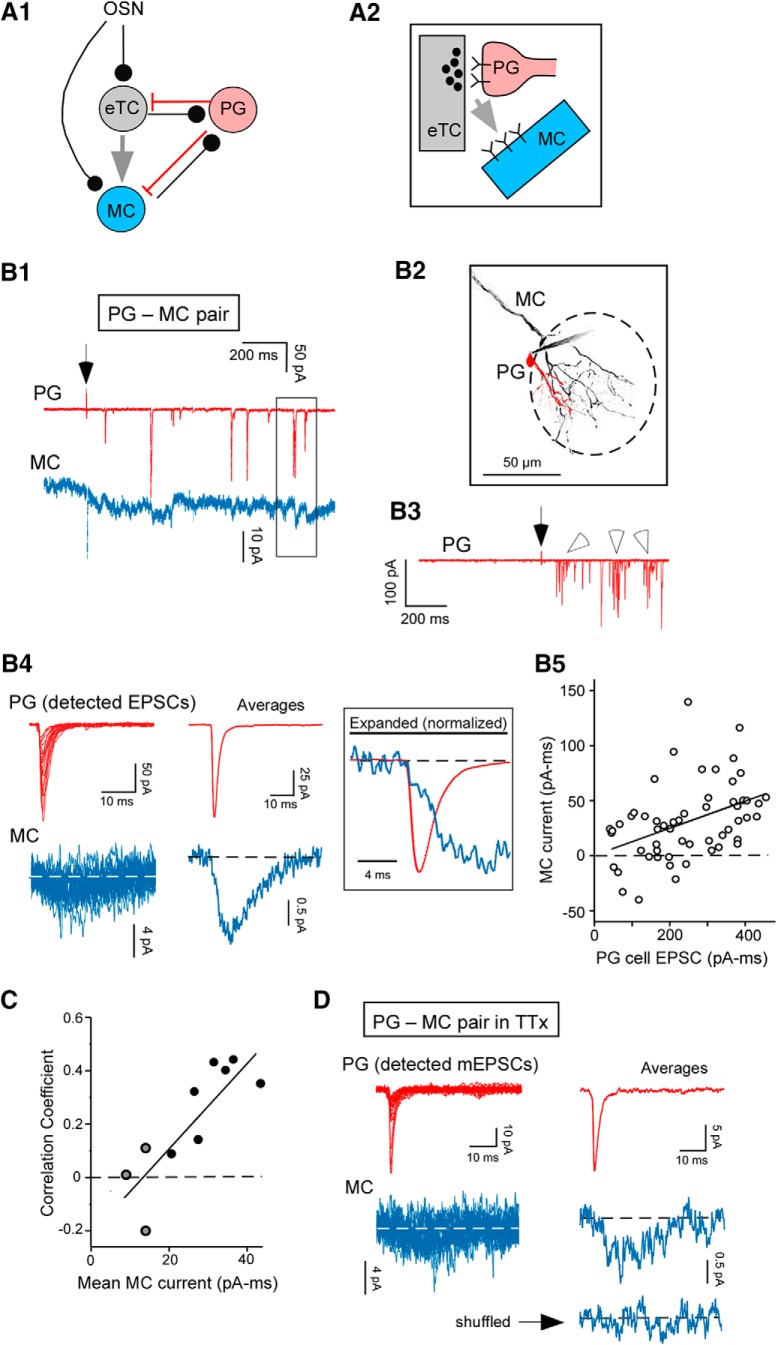
Tests of spillover hypothesis based on PG cell-MC pair recordings. ***A1***, Simplified circuitry at a glomerulus: OSN axons contact eTCs (black lines), which in turn send glutamatergic extrasynaptic signals to MCs (gray arrow). eTCs and MCs can excite GABAergic PG cells at dendrodendritic synapses, which feedback inhibition onto these cells (red lines). The OSN-to-eTC-to-MC feedforward pathway occurs in parallel with the direct OSN-to-MC pathway (left). ***A2***, The spillover hypothesis: glutamate released at an eTC-to-PG cell synapse activates extrasynaptic receptors on nearby MC apical dendrites. ***B***, Example whole-cell current recordings in a same-glomerulus PG cell-MC pair (*V_hold_* = –77 mV in both cells) used to test the spillover hypothesis. Shown are currents evoked by OSN stimulation (40 µA) in a single response-trial (***B1***; PG cell in red, MC in blue), a fluorescent image of the pair (***B2***; glomerulus demarcated by dashed oval), five superimposed trials for the PG cell on a less expanded scale (***B3***), detected rapid EPSCs in the PG cell and time-locked MC currents (***B4***; expanded and normalized in inset), and a plot showing the correlation between individual PG cell and MC current events (***B5***; correlation coefficient = 0.43, *p* = 0.0010). Boxed region in ***B1*** shows two examples of current deflections in the MC that were time-locked to rapid EPSCs in the PG cell. Open arrowheads in ***B3*** point to bursts of EPSCs in the PG cell that delineate the cell as the subtype that receives direct input from eTCs (Shao et al., 2009). ***C***, Summary of correlation coefficients obtained across all PG cell-MC pair recordings, plotted as a function of the mean MC current in the same experiment (*r* = 0.78, *p* = 0.008). Plot combines data from our standard recordings (*n* = 7; black circles) as well as three recordings in TTx (see ***D***, gray circles). ***D***, Spontaneous currents in a PG cell-MC pair recorded in TTx (1 µM), consistent with spillover at single release sites. The bottom trace at right reflects the average of shuffled events collected from the MC. Averages reflect 22 events.

**Figure 2.**
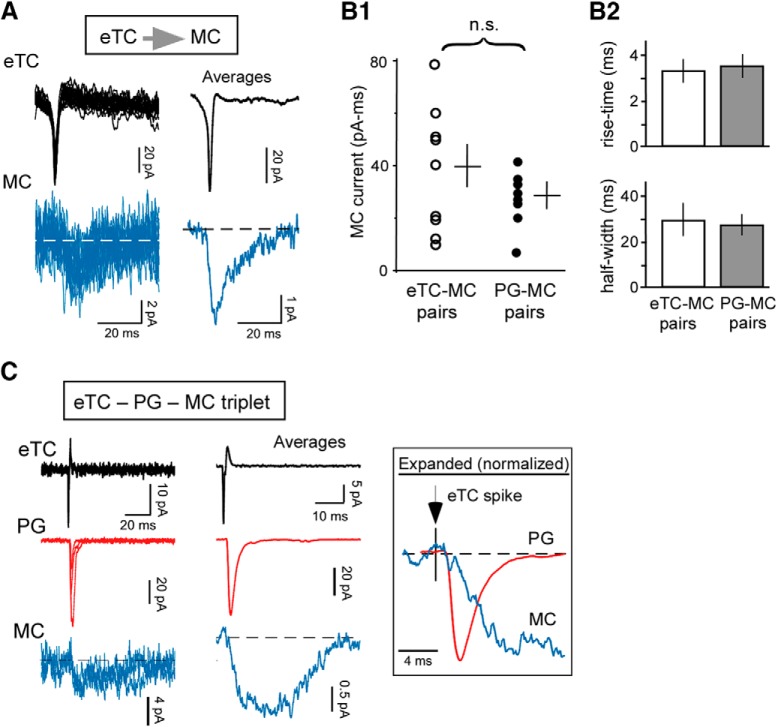
Spillover-mediated currents are due to glutamate release from eTCs. ***A***, Recording from an eTC-MC pair showing MC currents (blue; at *V_hold_* = –77 mV) evoked by single eTC spikes (black; in LCA mode). Raw traces (left) and averages (*n* = 94) are shown. Note the amplitude and kinetic similarities to MC currents recorded in the PG cell-MC pairs (Fig. 1*B4*). ***B***, Comparison of the magnitude (***B1***) and kinetic properties (***B2***; 20%-to-80% rise-time and half-width) of MC currents recorded in eTC-MC pairs (*n* = 9) versus PG cell-MC pairs (*n* = 8 for ***B1***, *n* = 7 for ***B2***). Lines in ***B1*** reflect mean ± SEM. Integrated charge values were multiplied by –1. ***C***, Current events collected from a triple-cell recording that included a same-glomerulus eTC, PG cell, and MC. In the expanded and normalized average traces (boxed inset), it is clear that the PG cell and MC currents both had 1- to 2-ms onset delays after the eTC spike, indicating that the eTC was the source of the currents.

One locus where extrasynaptic glutamate plays a prominent role is the mammalian olfactory bulb. Within glomeruli, the dendrites of different output mitral cells (MCs) can release glutamate and excite other MCs (Isaacson, 1999; Schoppa and Westbrook, 2002; Urban and Sakmann, 2002; Pimentel and Margrie, 2008) in the apparent absence of dendrodendritic chemical synaptic connections (Pinching and Powell, 1971; Bourne and Schoppa, 2017). More recent studies have indicated that extrasynaptic transmission onto MCs also occurs from excitatory interneurons known as external tufted cells (eTCs; De Saint Jan et al., 2009; Najac et al., 2011; Gire et al., 2012). The extrasynaptic eTC-to-MC signals are noteworthy since they appear to be part of a multi-cellular, feedforward pathway of information flow from olfactory sensory neurons (OSNs) to MCs (OSN-to-eTC-to-MC) that exists in parallel with direct OSN-to-MC signaling (Fig. 1*A1*; De Saint Jan et al., 2009; Najac et al., 2011; Gire et al., 2012; Vaaga and Westbrook, 2016). Depending on stimulus conditions, the OSN-to-eTC-to-MC pathway can be dominant, reflecting the fact that eTCs are easily excited by OSNs. A number of important questions however remain about extrasynaptic eTC-to-MC signaling, including its underlying mechanism. In addition, from a more functional perspective, a key question is how extrasynaptic excitation can compete with local inhibition within a glomerulus. The extrasynaptic currents in MCs generated by single action potentials in eTCs are quite weak, 1–2 pA in amplitude (Gire et al., 2012; Fig. 2*A1*), as might be expected for glutamatergic signals mediated by receptors at distant sites, but eTCs also synaptically excite GABAergic periglomerular (PG) cells (Hayar et al., 2004a) that function to suppress MC activity (Gire and Schoppa, 2009; Shao et al., 2012; Fukunaga et al., 2014; Geramita and Urban, 2017). The high concentration of glutamate at synaptic receptors, together with the high input resistance of PG cells (Hayar et al., 2004b; Murphy et al., 2005; Shao et al., 2009), should mean that PG cells are well excited by eTCs leading to robust inhibition.

Here, we have combined multi-cell patch-clamp recordings in rat olfactory bulb slices and ultrastructural approaches to investigate extrasynaptic glutamatergic signaling within glomeruli. In mechanistic studies, we first provide evidence that extrasynaptic eTC-to-MC signaling not only cooccurs with eTC-to-PG cell synaptic transmission, but in fact results from spillover of glutamate released at eTC-to-PG cell synapses. Subsequently, we established quantitative relationships between stimulus strength and extrasynaptic excitation versus synaptic excitation of PG cells and the resulting inhibition. Our results show that extrasynaptic glutamatergic transmission is indeed much weaker than synaptic excitation and inhibition with weak stimuli, but can overcome its inherent disadvantages with respect to inhibition by displaying supralinear increases with repeated stimuli. This differs from sublinearities observed for inhibition. The shifting balance between extrasynaptic excitation and inhibition with stimulus strength could underlie an intraglomerular thresholding mechanism previously hypothesized to exist based on computational (Cleland and Sethupathy, 2006) and *in vivo* (Fukunaga et al., 2014) studies.

## Materials and Methods

### Animals and slice preparation

Male and female 8- to 20-d-old Sprague Dawley rats obtained from Charles River Laboratories were used. All experiments were conducted under protocols approved by the Animal Care and Use Committee of the University of Colorado, Anschutz Medical Campus.

Acute horizontal olfactory bulb slices (300–400 μm) were prepared following isoflurane anesthesia and decapitation. Olfactory bulbs were rapidly removed and placed in oxygenated (95% O_2_, 5% CO_2_) ice-cold solution containing the following: 72 mM sucrose, 83 mM NaCl, 26 mM NaHCO_3_, 10 mM glucose, 1.25 mM NaH_2_PO_4_, 3.5 mM KCl, 3 mM MgCl_2_, and 0.5 mM CaCl_2_ adjusted to 295 mOsm. Olfactory bulbs were separated into hemispheres with a razor blade and attached to a stage using adhesive glue applied to the ventral surface of the tissue. Slices were cut using a vibrating microslicer (Leica VT1000S) and were incubated in a holding chamber for 30 min at 32°C. Subsequently, the slices were stored at room temperature.

### Electrophysiological recordings

Experiments were conducted under an upright Zeiss Axioskop2 FS Plus microscope (Carl Zeiss MicroImaging) fitted with differential interference contrast (DIC) optics, video microscopy and a CCD camera (Hamamatsu). Identified cells were visualized with 10× or 40× Zeiss water-immersion objectives. Recordings were performed at 32–35°C.

The base extracellular recording solution contained the following: 125 mM NaCl, 25 mM NaHCO_3_, 1.25 mM NaHPO_4_, 25 mM glucose, 3 mM KCl, 1 mM MgCl_2_, and 2 mM CaCl_2_ (pH 7.3 and adjusted to 295 mOsm), and was oxygenated (95% O_2_, 5% CO_2_). The pipette solution for most whole-cell recordings contained the following: 125 mM K-gluconate, 2 mM MgCl_2_, 0.025 mM CaCl_2_, 1 mM EGTA, 2 mM Na_3_ATP, 0.5 mM Na_3_GTP, and 10 mM HEPES (pH 7.3 with KOH, osmolarity adjusted to 215 mOsm). For whole-cell recordings from eTCs, 30 mM glutamic acid was added to the pipette to prevent run-down of evoked glutamatergic currents (Ma and Lowe, 2007). For whole cell recordings of eTC and MC current responses to OSN stimulation, the K-gluconate in the pipette solution was replaced with an equimolar amount of cesium methanosulfonate, as well as the sodium channel blocker QX-314 (10 mM) to block action potentials. All whole-cell recordings included 100 μM Alexa Fluor 488 or Alexa Fluor 594 in the pipette solution to allow for visualization of cell processes. Loose cell-attached (LCA) recordings from eTCs were made with a pipette that contained the extracellular solution. Patch pipettes, fabricated from borosilicate glass, were pulled to a resistance of 3–4 MΩ for MCs, 4–6 MΩ for eTCs, and 6–8 MΩ for PG cells. Current and voltage signals in the single- and pair-cell experiments were recorded with a Multiclamp 700B amplifier (Molecular Devices), low-pass filtered at 1.8 kHz using an eight-pole Bessel filter, and digitized at 10 kHz. Triple-cell recordings also incorporated an Axopatch 200B amplifier (Molecular Devices). Data were acquired using Axograph X software on an Apple Mac Pro computer. All drugs were delivered via bath application at a flow rate constant to the baseline measurements.

Cell identity was determined in part by visualizing Alexa Fluor 488-mediated or Alexa Fluor 594-mediated fluorescence signals. MCs were easily identified by their position in the MC layer and large cell bodies. eTCs were distinguished from other cells in the glomerular layer by their position in the inner half of the layer, their relatively large, spindle-shaped somata (≥10 μm in diameter), a single highly branched apical dendrite and no lateral dendrite, and a relatively low input resistance (∼0.2 GΩ; Hayar et al., 2004b). PG cells were identified by their small soma (<10 μm in diameter), small dendritic arbor that was confined to one glomerulus, and high input resistance (>0.5 GΩ; Hayar et al., 2004b; Murphy et al., 2005; Shao et al., 2009). In addition, we only considered PG cells that displayed bursts of EPSCs reflecting inputs from bursting eTCs (∼70% of the total; Hayar et al., 2004a; Shao et al., 2009), either spontaneously or in response to OSN stimulation. Fluorescence measurements were performed under whole-field epi-illumination using a DG-4 light source (Sutter Instruments). Signals were detected by a CoolSnap II HQ CCD camera (Photometrics) under control of Slidebook (Intelligent Imaging Innovations) software.

In multi-cell recordings, we determined that the cells sent their apical dendrites to the same glomerulus in part based on anatomic measurements. Physiologic evidence that eTC-MC pairs were associated with the same glomerulus was obtained from the presence of perfectly coincident long-lasting depolarizations (LLDs; Carlson et al., 2000; Gire and Schoppa, 2009) when the two cells were in whole-cell patch mode. When an eTC was in LCA patch mode, LLDs in the MC perfectly cooccurred with long-lasting bursts of spikes in the eTC. For recordings from eTC-PG cell pairs affiliated with the same glomerulus, every burst of spikes in the eTC was associated with a burst of rapid EPSCs in the PG cell (Hayar et al., 2004a) and no bursts of EPSCs in the PG cell were observed without cooccurring bursts of spikes in the eTC.

For experiments employing OSN stimulation, stimulation of OSN axons was performed using a broken-tip patch pipette (5–10 μm in diameter) placed in the olfactory nerve layer, 50–100 μm superficial to the glomerular layer. Current injections were delivered by a stimulus isolator (World Precision Instruments) under control of a TTL output from Axograph X software. Weak intensities of electrical stimulation were used (1–50 µA) and test eTCs and MCs were chosen that were associated with glomeruli at the surface of the slice. This enabled us to stimulate OSNs at one glomerulus with little or no activity in neighboring glomeruli (McGann et al., 2005; Gire and Schoppa, 2009). Stimulus artifacts in many of the illustrated traces have been blanked or truncated.

In most whole-cell recordings, the osmolarity of the patch pipette solution was adjusted to a relatively low value of 215 mOsm (see above), as we found that this assisted patch formation and stability. To address a potential concern that the low osmolarity artificially increased extrasynaptic glutamatergic signaling, we recorded extrasynaptic currents in MCs that were evoked by OSN stimulation (Gire et al., 2012) using either the low-osmolarity pipette solution or a 290 mOsm solution. No significant differences were observed in the magnitude of the slow current (integrated charge = 8.9 ± 2.2 pA/ms for 215 mOsm, *n* = 6; 6.0 ± 0.8 pA/ms for 290 mOsm, *n* = 5; *p* = 0.22 in unpaired *t* test) nor in the enhancing effect of the glutamate transport blocker DL-TBOA (10–50 µM; 92 ± 26% increase in integrated charge for 215 mOsm, *n* = 6; 142 ± 47% increase for 290 mOsm, *n* = 5; *p* = 0.42 in unpaired *t* test). Thus, the predominance of extrasynaptic transmission in MCs did not appear to be an artifact of the low osmolarity pipette solution. We also tested whether the depressing form of eTC-to-PG cell transmission that was observed during burst events recorded with a 215 mOsm solution also occurred with a 290 mOsm pipette solution applied to the PG cell. No difference in depression was found, as quantified by the amplitude ratio for the second versus first EPSCs in spontaneous burst events (0.45 ± 0.05 for 215 mOsm, *n* = 7; 0.34 ± 0.04 for 290 mOsm, *n* = 4; *p* = 0.17, unpaired *t* test).

### Experimental design and statistical methods

Data were analyzed using Axograph or Microsoft Excel and are generally expressed as mean ± SEM (except where noted). Significance was most commonly determined using two-tailed non-parametric tests, either the Wilcoxon matched-pairs signed-rank test or the Mann–Whitney *U* test. Paired *t* tests were used (as indicated) in a number of matched pair comparisons when there was evidence for normality in the distribution of the data values or due to a smaller sample size (*n* = 5 or 6). The Kolmogorov–Smirnov test was used to compare the distributions of MC current magnitudes in the eTC-MC pairs, with and without LLD events. A value of *p* < 0.05 was considered significant (asterisks in the figures), except if multiple comparisons were made, in which case the Bonferroni correction was applied.

### Analysis of electrophysiological data

In the MC-PG cell pair recordings used for determining the mechanisms of eTC-to-MC extrasynaptic transmission, an event detection routine was used to detect rapid EPSCs [or miniature EPSCs (mEPSCs) in the presence of tetrodotoxin (TTx)] in the PG cell and the cooccurring current in the MC was also captured. We considered there to be a significant time-locked current in the MC if the cooccurring average current in the MC (measured 5–25 ms after the start of the EPSC in the PG cell) was >2 times the standard deviation of the averaged time-shuffled current. Analysis of MC-PG cell currents was conducted most commonly on responses to OSN stimulation (*n* = 5), and, in some cases (*n* = 3), on spontaneous current events. All of the PG cells analyzed displayed complex currents that included bursts of EPSCs that are characteristic of the ∼70% of PG cells that are excited by eTCs (Hayar et al., 2004a; Shao et al., 2009). However, we focused here on isolated EPSC events in the PG cells (≥50-ms event separation) because of the requirement for precise temporal information in our analysis. In the triple-cell recordings, isolated action potential currents (>50-ms separation from other spikes) were detected in the eTC using a thresholding method, and currents locked to these spikes were averaged in the PG cell and MC. In the eTC-MC pair recordings used to test for spillover at excitatory axonal synapses on eTCs, a similar analysis to that used with the PG-MC pairs was performed, except rapid, spontaneously-occurring EPSCs in the eTC were first detected.

In the pair-cell recordings used to analyze the relationship between eTC spike number and *I_extra_*, current integrals were measured over varying time-windows (50–700 ms) reflecting differences in the apparent duration of *I_extra_* for different numbers of eTC spikes. From the measurements of charge associated with *I_extra_*, we computed a parameter *S_N_* that reflected the deviation from linearity in the rise of the current for increasing spike number (*N*) from:

1 $$S_{N}=I_{extra(N)}/\left(N\text{x}I_{extra(1\;spike)}\right)$$

In this expression, *I_extra(N)_* was the magnitude of the extrasynaptic current when the eTC engaged in *N* spikes, while *I_extra(1 spike)_*was the extrasynaptic current evoked when the same eTC spiked once. Linearity, corresponding to when *S_N_* = 1, occurred when the observed extrasynaptic current was the linear summation of the current evoked by one spike. For the recordings from eTC-PG cell pairs, a similar analysis was performed based on integrating the bursts of rapid EPSCs in the PG cell for eTCs engaged in *N* spikes. In the assessment of whether PG cells displayed slow, extrasynaptic currents, spontaneous EPSC (sEPSC) bursts in PG cells that displayed more than or equal to four rapid EPSCs were selected for each recording, aligned to the start of the first EPSC in each burst, and then averaged. Currents were then integrated in a 100-ms window, 100–200 ms after the start of the first EPSC. In the initial selection of the rapid EPSC bursts before averaging, those that persisted for longer than 100 ms were excluded.

In the analysis of the eTC-PG cell pair recordings testing for recurrent excitation, action potential currents in the eTC were first detected using a thresholding method and 16-ms intervals of PG cell current centered on the time of each eTC spike were selected. Rapid EPSCs were then detected using an amplitude threshold on filtered (600–800 Hz) derivatives of the current. The threshold was selected based on visual inspection of whether the procedure effectively detected EPSCs in the PG cell with no false positives. Detected EPSCs that occurred in a window 0.5–3.5 ms after the eTC spike were considered to reflect direct transmission from the test eTC to the test PG cell. Because in our primary analysis we wished to exclude eTC spike bursts that were associated with LLDs, eTC spike bursts were initially sorted by whether there were cooccurring LLDs. Information was available about the LLD from either a simultaneous MC recording (in two triple-cell recordings) or from the eTC recording itself (when the eTC recording was in current-clamp mode).

In measurements of excitatory and inhibitory currents in eTCs in response to OSN stimulation, different holding potentials (–77 and +28 mV, respectively) were used to isolate each current type. At +28 mV, some of the outward current in principle could have reflected glutamatergic currents with reversed polarity, not just a GABAergic current. This possibility however was excluded by comparing the time courses of the eTC currents at +28 and –77 mV just after OSN stimulation. When the OSN-EPSC peaked at –77 mV (∼3 ms after OSN stimulation), the currents at +28 mV were either near zero or small inward deflections, not an outward current. Prior studies also indicated that outward currents evoked by OSN stimulation near +28 mV were blocked by the GABA_A_ receptor blocker gabazine (Zak et al., 2015). The evaluation of *I_extra_* from the eTC current recorded at –77 mV required reliable isolation of *I_extra_* from the monosynaptic OSN-EPSC using a subtraction procedure. With strong OSN stimulation, isolation of *I_extra_* became difficult in some recordings as *I_extra_* blended in with the OSN-EPSC. Evoked excitatory currents in an eTC were excluded from further analysis if the time constant of the exponential function fitted to the decay of the OSN-EPSC (used for subtracting the OSN-EPSC) deviated by >10% from the value used to fit the OSN-EPSC at lower stimulation intensities.

### Ultrastructural studies

The tissue sections from the olfactory bulb used for electron microscopy analysis were the same as those from Bourne and Schoppa (2017). The methods of preparing olfactory bulb slices used for filling eTCs with biocytin, tissue fixation, DAB labeling, and the final slicing of thin (50 nm) sections before EM analysis, are described in that study. Sections were imaged either on a FEI Tecnai G2 transmission electron microscope at 80 kV with a Gatan UltraScan 1000 digital camera at a magnification of 4800× or a Zeiss SUPRA 40 field-emission scanning electron microscope (FE-SEM) equipped with an integrated module called ATLAS (Automated Large Area Scanning; software version 3.5.2.385; Kuwajima et al., 2013).

The serial section images were aligned and dendrites, axons, and glia were traced using the RECONSTRUCT software (available for free download at http://synapses.clm.utexas.edu; Fiala and Harris, 2001; Fiala, 2005). For this study, we analyzed the neuropil surrounding dendrodendritic synapses between eTC dendrites and putative inhibitory dendrites as well as OSN synapses onto eTC dendrites. Although the dark precipitate formed by the DAB reaction obscured the interior of the eTC dendrite, presynaptic vesicles could still be identified and all analyzed dendrodendritic synapses had a clear asymmetric postsynaptic density. Similarly, OSN synapses onto DAB-labeled eTC dendrites were identified based on docked presynaptic vesicles, a clear synaptic cleft, and often a visible asymmetric postsynaptic density. Identification of the surrounding elements of the neuropil was based on their ultrastructural features. Glial processes were identified based on the presence of a dark cytoplasm (likely due to endogenous peroxidase activity that reacted with the DAB; Schipper et al., 1990), absence of vesicles, and long, thin morphology (Ventura and Harris, 1999), with processes tapering to <20 nm in some sections. Excitatory dendrites had round, clear vesicles and formed asymmetric synapses. Inhibitory dendrites had flattened, pleiomorphic vesicles and formed symmetric synapses. Dendrites were distinguished from axons based on their lack of boutons, fewer vesicles, and the presence of efferent and afferent synapses. Dendritic processes that formed at least one gap junction with another dendrite were assigned to be putative MC dendrites. This assignment was based on prior evidence that MC dendrites form many more gap junctions than eTCs (Hayar et al., 2005; Gire et al., 2012).

Each of the eTC-to-PG cell synapses (13) and OSN-to-eTC synapses (39) was examined through serial sections to determine whether glial processes or putative MC dendrites were nearby, within 0.5 µm. This number, 0.5 µm, was chosen to be in the range of extrasynaptic transmission in part based on the modeling studies of Pendyam et al. (2009), which showed that supralinear increases in glutamate concentration could occur at distances from glutamate release sites near this value.

A subset (10) of the eTC-to-PG cell synapses were selected for a brick analysis to further characterize the surrounding neuropil. A 2 × 2 μm grid was positioned with the synapses in the middle. Depending on the size of the synapses, the grid was copied to the same location on serial sections above and below the synapses to sample the surrounding neuropil, for a total of five bricks spanning 14–20 sections (50 nm/section; some bricks had more than one eTC-to-PG cell synapse). Each structure (astroglia, putative excitatory dendrite, putative inhibitory dendrite, axon) contained within the brick was identified by following it through serial sections.

## Results

Extrasynaptic transmission from glutamatergic eTCs onto MCs (De Saint Jan et al., 2009; Najac et al., 2011; Gire et al., 2012) is part of a multi-step feedforward pathway of excitation between OSNs and MCs (OSN-to-eTC-to-MCs; Fig. 1*A1*). Our main objectives were (1) to examine the underlying mechanism of eTC-to-MC extrasynaptic signaling and (2) to evaluate the magnitude of eTC-to-MC extrasynaptic excitation versus synaptic inhibition under varying conditions of stimulus strength. A premise for this study, that eTC-to-MC signaling occurs extrasynaptically, is based on prior morphologic evidence that eTCs make few if any direct synaptic contacts on MCs (Pinching and Powell, 1971; Bourne and Schoppa, 2017). In addition, eTC-to-MC currents are selectively attenuated by a low-affinity AMPA receptor antagonist (Gire et al., 2012). This assumption will be revisited in the Discussion as we discuss our mechanistic findings in the context of new experimental results presented here.

### eTC-to-MC transmission is due to spillover of glutamate at eTC-to-PG cell synapses

Our primary hypothesis for the mechanism of eTC-to-MC transmission (Fig. 1*A2*) was that it reflected glutamate released at eTC synapses onto GABAergic PG cells (Hayar et al., 2004a) diffusing (“spilling over”) out of the synaptic cleft and directly activating extrasynaptic glutamate receptors on MC apical dendrites (Salin et al., 2001). To test this spillover hypothesis, simultaneous voltage-clamp recordings were first performed in cell-pairs that included a MC and a PG cell affiliated with the same glomerulus (*V_hold_* = –77 mV in both cells; example in Fig. 1*B1–B5*). The presence of slow excitatory currents in MCs that were precisely time-locked to rapid AMPA receptor-mediated EPSCs in PG cells that reflected direct eTC-to-PG cell transmission would provide evidence for spillover. In most experiments, we examined PG cell and MC currents following electrical stimulation of OSNs (100-µs pulse at 5–44 µA). This stimulation resulted in sustained excitation of the glomerular network that lasted hundreds of milliseconds (Schoppa and Westbrook, 2001; Yuan and Knöpfel, 2006; De Saint Jan and Westbrook, 2007) and bursts of EPSCs in the PG cells (Fig. 1*B3*) that reflected spike bursts in eTCs (Hayar et al., 2004b). Hence, the test PG cells were clearly in the subgroup of PG cells that receive direct synaptic inputs from eTCs (Hayar et al., 2004a; Shao et al., 2009).

We found clear evidence for potential spillover in seven out of eight PG cell-MC pairs, as evidenced by the presence of time-locked MC and PG cell currents (mean onset latency between averaged PG cell and MC currents = 0.2 ± 0.1 ms, *n* = 7; Fig. 1*B1*,*B4*). Furthermore, the MC current events were correlated in size to their associated PG cell EPSCs (mean correlation coefficient *r* = 0.31 ± 0.05, *p* < 0.05 in Wilcoxon matched-pairs signed-rank test in comparison to *r* = 0 across seven pairs; *p* ≤ 0.014 in five of seven pairs when coupled currents were analyzed by experiment; Fig. 1*B5*). Such correlations would be expected if the MCs and PG cells were responding to the same bolus(es) of glutamate, which should naturally vary in magnitude due to variability in the amount of glutamate in a vesicle and/or the number of vesicles released per action potential. The correlation coefficients were modest, but this likely reflected the small size and low signal-to-noise of the MC currents. Indeed, consistent with this explanation, we found that the values of the correlation coefficients across the pairs were themselves positively correlated to the mean MC current (*r* = 0.78, *p* = 0.008; Fig. 1*C*).

Additional evidence for spillover was obtained in PG cell-MC pair recordings conducted in the presence of the sodium channel blocker TTx (1 µM; Fig. 1*D*). In three of seven same glomerulus-pairs, small MC currents were observed (mean integrated charge = –12 ± 2 pA/ms, *n* = 3) that were time-locked to spontaneous mEPSCs in PG cells (mean peak amplitude = –12 ± 1 pA, mean current onset-latency between PG cell and MC currents = 0.4 ± 0.3 ms, *n* = 3). Because spontaneous glutamate release in TTx should generally be occurring at single release sites at a time, the slow MC currents locked to the mEPSCs likely resulted from glutamate release at the same sites.

The presence of time-locked currents in the PG cell-MC pairs were consistent with the spillover hypothesis for eTC-to-MC transmission (Fig. 1*A2*), but what evidence was there that the PG cell and MC currents resulted from glutamate release from eTCs? To address this question, we first compared the properties of the MC currents recorded in the PG cell-MC pairs to MC currents evoked by single eTC spikes in eTC-MC pairs (Fig. 2*A*). We found that the MC currents in the two sets of recordings were indistinguishable, with similar magnitude and kinetic properties (MCs in PG-MC pairs: integrated charge = –31 ± 5 pA/ms, *n* = 8; 20–80% rise-time = 3.3 ± 0.5 ms, *n* = 7; half-width = 29 ± 7 ms, *n* = 7; MCs in eTC-MC pairs: integrated charge = –39 ± 8 pA/ms, 20–80% rise-time = 3.6 ± 0.5 ms, half-width = 27 ± 4 ms, *n* = 9 for all variables; *p* > 0.10 in Mann–Whitney *U* test comparing all variables; Fig. 2*B*). This suggested that the MC currents in the PG cell-MC pairs were derived from the same source as the currents in the eTC-MC pairs. Direct evidence that eTCs drove coupled currents in PG cells and MCs was also obtained in two triple-cell recordings that included MCs, PG cells, and eTCs at the same glomerulus (example in Fig. 2*C*). In these, the MCs displayed small currents (integrated charge of averaged currents = –42 and –20 pA/ms) that were time-locked to both rapid EPSCs in the PG cell and spikes in the eTC.

We also excluded potential alternate sources for the MC currents in the PG cell-MC pair recordings. For example, the possibility that the currents reflected glutamate release from OSNs was excluded by the fact that, in the recordings that involved OSN stimulation, the first 50 ms of the response after OSN stimulation was avoided in the analysis. Glutamate release at MC-to-PG cell synapses also could not have contributed to the time-locked currents that we measured. In the OSN stimulation experiments, weak stimuli were used that never elicited the well-characterized recurrent excitatory events in MCs known as LLDs (Carlson et al., 2000; Figs. 4*E1*, 5*A3*). Because prior studies have shown that delayed spiking in MCs following single OSN stimulus pulses depends on LLDs being generated (Gire and Schoppa, 2009), action potential-driven glutamate release from MCs could not have contributed to the currents in the PG cell-MC pairs that we analyzed.

**Figure 3.**
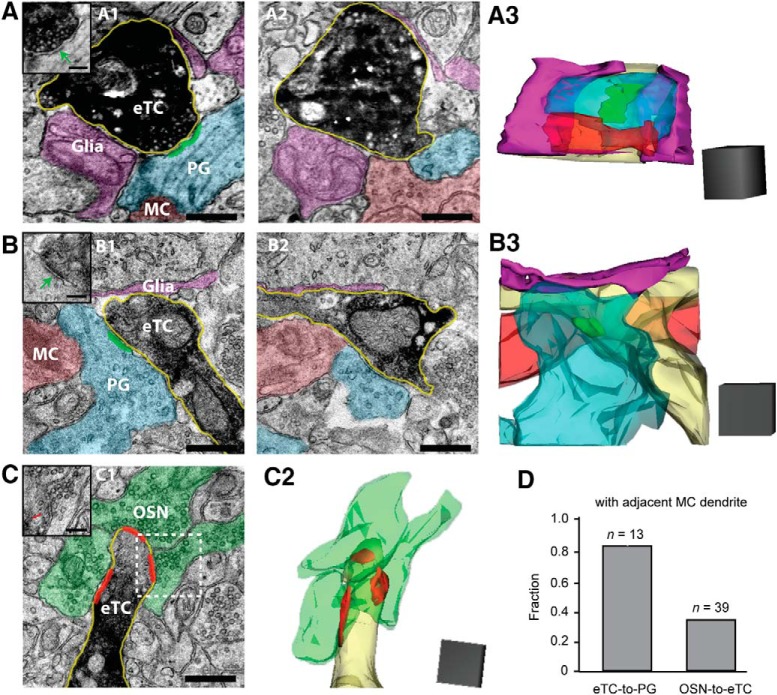
Ultrastructural evidence for complexes that could support spillover. ***A***, Electron micrographs (***A1***, ***A2***; two images are four 50-nm sections apart) and three-dimensional reconstruction (***A3***) of an example complex that includes a DAB-labeled eTC dendrite (darkened in micrographs; light yellow in reconstruction) forming a synapse (green) onto a putative PG cell dendrite (blue). A glutamatergic dendrite that was assigned to be a putative MC (red; see main text) and glial processes (purple) are in close proximity. Note that the glial processes in the reconstruction appear to surround the dendrites. Inset in ***A1***, Same image as in ***A1*** but without the colors so that the synapse (green arrow) can be seen more clearly. Scale bars: 0.5 μm for micrographs, 0.1 μm for inset of ***A1***. Scale cube in ***A3*** = 0.5 µm^3^. ***B***, Another example of a complex containing an eTC-to-PG cell synapse with adjacent glial process and putative MC dendrite. Note that in the reconstruction the putative MC dendrite runs behind the PG cell and eTC dendrites. Scale bars and cube as in ***A***. ***C***, Electron micrograph (***C1***) and reconstruction (***C2***) of a cluster of synapses (in red) from OSNs (green) onto a DAB-labeled eTC dendrite (light yellow in reconstruction). Note the absence of an adjacent putative MC dendrite. Inset in ***C1***, Original image of the eTC dendrite showing one of the OSN synapses (red arrow) that is indicated by the white dashed box. Scale bars and cube as in ***A***. ***D***, Summary of analysis of 13 eTC-to-PG cell synapses and 39 OSN-to-eTC synapses (pooled results from analysis of two eTC fills in two bulb slices). The fraction out of the total with an adjacent putative MC dendrite (within 0.5 µm) was much higher for eTC-to-PG cell synapses.

Finally, as an independent check on the spillover hypothesis, ultrastructural studies were conducted on olfactory bulb slices in which eTCs were labeled with biocytin during whole-cell patch recordings (Bourne and Schoppa, 2017; Fig. 3). In reconstructions based on electron micrographs, we found ample evidence for complexes that could underlie spillover at eTC-to-PG cell synapses. Among 13 identified dendrodendritic synapses from eTC cells onto inhibitory dendrites (mostly presumed to be PG cells), 11 had nearby glutamatergic dendrites (within 0.5 µm; two examples in Fig. 3*A1–A3*,*B1–B3*; Fig. 3*D*). Furthermore, glial processes could be identified that extended close to the eTC-PG cell-glutamatergic dendrite complexes (in six of 13 synapses) and sometimes surrounded the complexes (Fig. 3*A3*). Importantly, the glial processes never surrounded the eTC-to-PG cell synapse, isolating it from the glutamatergic dendrite (*n* = 11). Because these processes and their associated transporters typically limit glutamate spillover at excitatory synapses (Asztely et al., 1997; Murphy-Royal et al., 2017), their absence would greatly facilitate the ability of glutamate released from eTCs to act on extrasynaptic glutamate receptors. The identity of the glutamatergic dendrites near the eTC-to-PG cell synapses was not definitively known. However, they were presumed to be MCs based on the presence of gap junctions, which MCs express at a higher level than other glutamatergic cell types (at least eTCs; Hayar et al., 2005; Gire et al., 2012).

### Absence of spillover at excitatory axonal synapses in glomeruli

**Figure 4.**
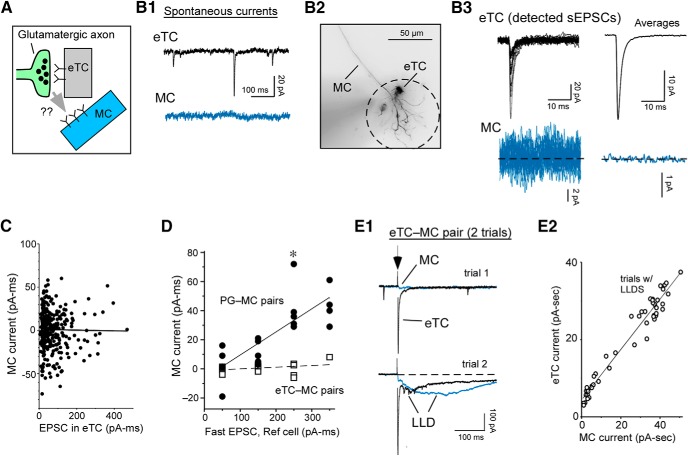
Absence of spillover at glutamatergic axonal synapses in glomeruli. ***A***, Model to be tested: glutamate released at axonal synapses onto eTC dendrites spills over and activates glutamate receptors on adjacent MC dendrites. ***B***, Example whole-cell recording from an eTC-MC pair at the same glomerulus used to test for spillover at axonal synapses. Raw traces recorded without a stimulus (***B1***), an image of the pair (***B2***; collapsed in the *z*-axis), and examples and averages (*n* = 293) of detected sEPSCs in the eTC and time-locked MC currents (***B3***) are illustrated. The cell body of the eTC in this pair was just above the glomerulus to which the MC sent its apical dendrite. ***C***, Plot relating the magnitude of the sEPSCs and associated MC currents for the experiment in ***B***. Line reflects linear regression fit of the data (*r* = –0.012, *p* = 0.82). ***D***, Summary of MC currents recorded in eTC-MC pairs (open squares) versus PG cell-MC pairs (filled circles), plotted as a function of the amplitude of the fast EPSC in the reference cell (either eTC or PG cell). Data were binned according to the magnitude of the fast EPSCs in 100-pA/ms increments. Note that, while the MC current in the PG cell-MC pairs clearly increased as a function of the magnitude of the PG cell EPSC (linear regression fit: *r* = 0.79, *p* = 5.6 × 10^−5^), no such relationship was observed in the eTC-MC pairs (*r* = 0.28, *p* = 0.31). The eTC-MC pair dataset includes four to five points each in the bins centered at 50, 150, and 250 pA. Each point in each bin reflects a single experiment; *p = 0.01 in Mann–Whitney *U* test comparing the MC current in the two cell-pair types. ***E***, Electrophysiological evidence that the eTC and MC in ***B*** were affiliated with the same glomerulus. ***E1***, Two response trials overlaid showing that OSN stimulation (38 µA; at arrow) evoked LLDs in both cells in trial 2, but not trial 1. ***E2***, Summary plot of responses to OSN stimulation for the same experiment (49 trials) relating the magnitude of the delayed current in the two cells. Note the clustering of data points in the upper right, corresponding to cooccurring LLD events. Current magnitude measurements were obtained by integrating the current starting 50 ms after OSN stimulation to avoid the OSN-EPSC. Line reflects linear regression fit (*r* = 0.98, *p* < 1.0 × 10^−6^).

Because glomeruli in the bulb are compartmentalized structures with confined spaces (Chao et al., 1997; Kasowski et al., 1999), we wondered whether glutamate spillover only occurs as a result of glutamate released at eTC-to-PG cell dendrodendritic synapses. To test for spillover at axonal glutamatergic synapses in glomeruli (Fig. 4*A*), we performed simultaneous pair-cell recordings in eTCs and MCs affiliated with the same glomerulus, similar to Figure 2*A*, but with both cells in voltage-clamp mode (*V_hold_*= –77 mV; Fig. 4*B*). In voltage-clamped eTCs, fast sEPSCs could be identified (Hayar et al., 2005) that reflect axonal synapses, either from OSNs or centrifugal inputs from cortical areas (Markopoulos et al., 2012; Rothermel and Wachowiak, 2014). Spillover at these axonal synapses should have been apparent as slow currents in the MC locked to the fast eTC EPSCs, but in fact we found no such current (coupled current magnitude = –0.2 ± 0.6 pA/ms integrated charge, *n* = 6; Fig. 4*B3*,*C*,*D*). The conclusion that no spillover occurs at axonal synapses onto eTCs was also supported by our ultrastructural data (Fig. 3*C1*,*C2*,*D*). In contrast to the eTC-to-PG cell synapses (Fig. 3*A*,*B*,*D*), which had presumed MC dendrites in close proximity in the large majority (85%) of examples examined, OSN axonal synapses onto labeled eTCs had nearby dendrites of presumed MCs relatively infrequently (38%; 15 out of 39 OSN-to-eTC synapses).

One potential caveat to our physiologic results arguing against spillover at axonal synapses was the fact that the sEPSCs in eTCs in the eTC-MC pairs were small, with typical magnitudes near 20 pA (mean peak amplitude = –21 ± 3 pA, *n* = 6). This compared to values that were ∼3-fold larger for the eTC-to-PG cell EPSCs in the PG cell-MC pairs (mean peak amplitude = –63 ± 15 pA, *n* = 8; *p* = 0.028 in unpaired *t* test). The small sEPSCs in eTCs suggested that the absence of coupled MC currents could have been because axonal synapses are relatively weak, making it difficult to detect spillover. However, we found no coupled MC currents in the eTC-MC pairs (–1 ± 2 pA/ms integrated charge, *n* = 4) even when we focused on a subset of larger sEPSCs in eTCs (200–300 pA/ms; *p* = 0.01 in Mann–Whitney *U* test comparing MC currents coupled to eTC vs PG cell EPSCs of the same amplitude; Fig. 4*D*). The absence of coincident current events in the eTC-MC pairs was also not because the cells were affiliated with different glomeruli. In these experiments, we used two independent criteria to confirm that the pairs were affiliated with the same glomerulus. These included the cells’ anatomy (Fig. 4*B2*) as well as the cooccurrence of LLD events in the eTC and MC in the current recordings under stimulus conditions in which there were numerous failures to elicit LLDs (Fig. 4*E*). LLDs are recurrent excitatory events that are synchronized among excitatory cells affiliated with the same but not different glomeruli (Schoppa and Westbrook, 2001; Gire and Schoppa, 2009). Thus, we took the absence of coupled currents in the eTC-MC pair recordings as strong evidence against spillover at axonal synapses.

### Dynamics of extrasynaptic and synaptic transmission with increasing eTC spike number

Having established that a major part of extrasynaptic glutamatergic transmission from eTCs onto MCs is mediated by spillover at eTC-to-PG cell dendrodendritic synapses, we next turned to the more functional question about its importance. A potentially significant issue is the small size of the MC extrasynaptic current versus cooccurring synaptic excitation of PG cells. In terms of integrated charge, we found that the eTC-to-MC extrasynaptic current (∼–40 pA/ms; Fig. 2*B1*) was a factor of 10 smaller than the PG cell EPSC evoked by single eTC spikes (–443 ± 83 pA/ms, *n* = 7; *p* < 0.01 in Mann–Whitney *U* test; examples in Figs. 1*B4*, 2*C*). How effective could eTC spiking be in exciting MCs if, for every bout of extrasynaptic eTC-to-MC excitation, there is much stronger excitation of inhibitory circuits? We reasoned that one answer to this question could be that the relative level of eTC-driven extrasynaptic excitation of MCs versus synaptic excition of PG cells changes markedly when eTCs engage in multiple spikes. Studies in other systems have indicated that extrasynaptic glutamatergic currents exhibit large non-linearities with increasing stimulus strength or number that may reflect non-linear changes in local glutamatergic signaling at extrasynaptic sites (Carter and Regehr, 2000; Clark and Cull-Candy, 2002; Chalifoux and Carter, 2011; Nahir and Jahr, 2013).

**Figure 5.**
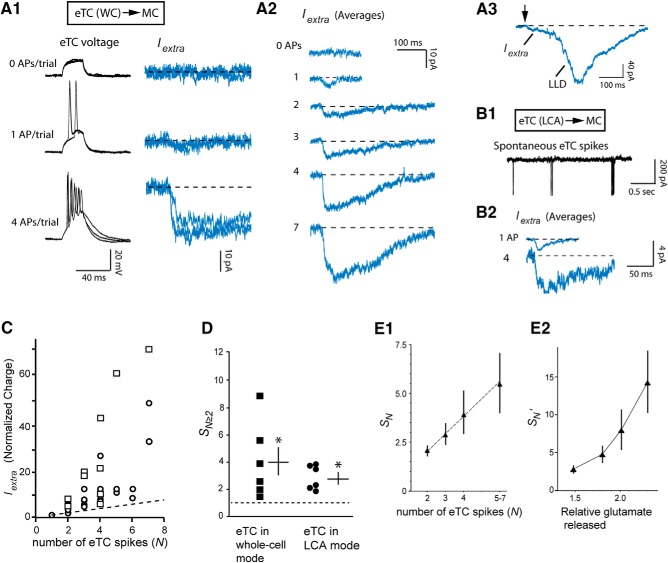
Supralinear increase in extrasynaptic excitatory currents. ***A***, Example whole-cell recordings from an eTC-MC pair illustrating MC current responses to different numbers of action potentials (APs) in the eTC. eTC spikes were evoked by direct depolarizing current pulses (500 pA, 25 ms). Note in the raw traces (***A1***; three superimposed trials) and averages (***A2***) that the total current associated with *I_extra_* for more than or equal to two eTC spikes was much larger than linear summation of the current driven by one AP. In one of the trials in which the eTC spiked seven times in the same experiment (***A3***), the MC response included a distinct, much larger LLD event ∼150 ms after the onset of *I_extra_* (note scale bar). ***B***, Another eTC-MC pair recording in which the number of spikes in the eTC recorded in the LCA mode (***B1***; raw example trace) was related to *I_extra_* in the MC (***B2***; averages). ***C***, Summary of *I_extra_* measurements from 12 pair recordings versus number of spikes in the eTC (*N*). Values, which reflect experiments in which the eTCs were in whole-cell (open squares) or LCA (open circles) patch modes, were normalized to the charge elicited by one eTC spike in the same pair. Note that nearly all points lie above the dashed line, which reflects linear summation of *I_extra_* elicited by one eTC spike. Most pair recordings contributed multiple data points to the plot. ***D***, Supralinearity indices *S_N_* measured across 12 pair-cell recordings separated by recording type for the eTC. A single value is plotted for each pair-cell recording, reflecting the average *I_extra_* measured whenever the eTC fired at least two spikes (*N* ≥ 2). Asterisks: *p* ≤ 0.041 in comparison to *S_N≥2_* = 1 (linear), paired *t* tests. Lines reflect mean ± SEM for each recording type. ***E1***, Supralinearity indices *S_N_* (mean ± SEM) sorted by the number of spikes in the eTC. Each data point reflects 8–10 recordings obtained when the eTC was in either whole-cell or LCA patch mode. Superimposed line reflects fit of the individual *S_N_* values across all experiments, which yielded a significant correlation coefficient (0.37, *p* = 0.031). ***E2***, Similar to part ***E1***, except that values for the supralinearity indices were adjusted (yielding *S_N_’*) for the relative amount of glutamate released across different numbers of eTC spikes. Glutamate release was estimated from EPSCs recorded in eTC-PG cell pairs (Fig. 6); *x*-axis values reflect estimates of the amount of glutamate release following two, three, four, or five to seven eTC spikes, normalized to the single spike-evoked release in the same recording.

Indeed, we found striking evidence for such non-linear behavior in the extrasynaptic currents recorded in our pair-cell recordings, referred to as *I_extra_* for the rest of ths study. In six pairs in which spiking in the eTC was controlled with direct current injection in whole-cell current-clamp mode, an increasing number of spikes in the eTC consistently increased *I_extra_* by much more than would be expected from linear summation of currents elicited by single eTC spikes (Fig. 5*A1*,*A2*,*C*). Defining a parameter *S_N_* as a measure of the degree of this “supra”-linearity for different spike numbers *N* (*S_N_* = 1 is linear; see Materials and Methods), we estimated that *I_extra_* driven by eTC spike bursts (*N* ≥ 2) was ∼4-fold larger than linear summation (*S_N_* = 4.0 ± 1.1, *n* = 6, *p* = 0.041 in paired *t* test in comparison to linear summation; Fig. 5*D*). Similar results were obtained in six additional pairs in which the eTC spiked while in LCA patch made (*S_N_* = 2.9 ± 0.4, *p* = 0.0026 in paired *t* test; Fig. 5*B1*,*B2*,*C*,*D*). The LCA recordings took advantage of the natural tendency of eTCs to engage in spontaneous spike bursts (Hayar et al., 2004b), while also enabling us to monitor the effect of eTC spiking without altering eTC physiology through changes in their intracellular content (Hayar et al., 2004b). Across the eTC-MC pair recordings, we also observed that the deviation from linearity grew with increasing spike number (*p* = 0.031 in linear fit of individual *S_N_* values across all eTC spike numbers more than or equal to two; Fig. 5*E1*) and that *I_extra_* evoked by the longest spike bursts (five to seven spikes) was, in total, 29 ± 9-fold larger than the current evoked by single spikes (*n* = 6). Importantly, the supralinearities did not simply reflect changes in the probability of global LLD events. Evoked LLDs, which were occasionally observed in the pair-cell recordings when the spiking eTC engaged in at least four spikes (Fig. 5*A3*), were easily distinguishable by size from *I_extra_* (*p* ≤ 0.027 from Kolmogorov–Smirnov tests of distributions of evoked current sizes in four pair-cell recordings).

**Figure 6.**
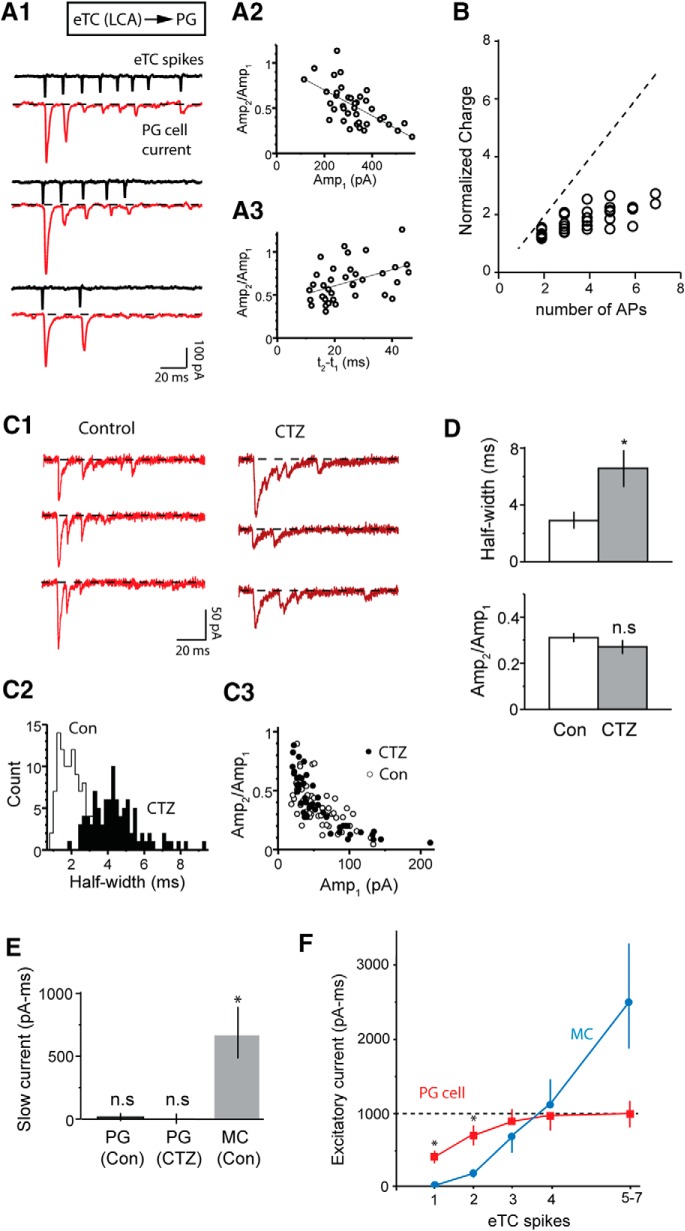
Sublinear increase in PG cell excitatory currents in eTC-PG cell pairs. ***A***, Example recordings in an eTC-PG cell pair showing that spontaneous bursts of spikes in the eTC (recorded in LCA mode) were associated with strongly depressing rapid EPSCs in the PG cell. Three selected examples with varying number of eTC spikes are illustrated in ***A1***. Analysis of 37 such bursts in this pair indicated that the degree of synaptic depression as reflected in the amplitude ratio of the first two EPSCs in the burst (*Amp_2_/Amp_1_*) was negatively correlated with the first EPSC amplitude (*r* = –0.63, *p* < 0.0001; ***A2***) and positively correlated with the interval between the first two EPSCs (*r* = 0.44, *p* = 0.0058; ***A3***). Both are consistent with a presynaptic vesicle depletion mechanism for depression. ***B***, Summary of integrated current measurements from 10 eTC-PG cell pair recordings, demonstrating that the synaptic depression resulted in a sublinear increase in the excitatory current as a function of spike number in the eTC. Diagonal line reflects linearity. ***C***, Example PG cell recording showing that the AMPA receptor allosteric modulator CTZ (100 µM) prolonged the EPSCs without altering the degree of depression. Selected EPSC bursts under each condition are illustrated in ***C1***, while EPSC half-widths (***C2***) and amplitude ratios (*Amp_2_/Amp_1_*; ***C3***) for the same experiment are also shown. The absence of an effect of CTZ on depression supported a presynaptic mechanism. ***D***, Histograms summarizing CTZ effects on EPSC half-width (top) and the *Amp_2_/Amp_1_* ratio (bottom) from five PG cell recordings; *p = 0.020 in paired *t* test. ***E***, Estimates of the magnitude of slow currents in PG cells not directly associated with rapid EPSCs. PG cell currents, both under control conditions and in CTZ, were measured in a 100- to 200-ms window after the start of each eTC spike burst for bursts with more than or equal to four spikes lasting <100 ms. MC currents (reflecting *I_extra_*) measured in the same manner are also plotted; *n* = 5 for each recording type; *p < 0.01 in paired *t* test comparison with zero current. ***F***, Summary of absolute charge measurements as a function of eTC spike number for *I_extra_* in MCs in the eTC-MC pairs (blue) and the PG cell current in the eTC-PG cell pairs (red). Each data point reflects mean ± SEM from 7–10 recordings; *p < 0.01 in Mann–Whitney *U* test, Bonferroni correction for multiple comparisons.

Glutamatergic extrasynaptic excitation of MCs rose in a highly supralinear fashion with eTC spike number, but synaptic excitation of GABAergic PG cells displayed markedly different properties (Fig. 6). In seven eTC-PG cell pair recordings, we found that the bursts of rapid EPSCs in the PG cell evoked by eTC spike bursts were characterized by strong depression (mean amplitude ratio of second vs first EPSCs, *Amp_2_/Amp_1_* = 0.45 ± 0.05; Fig. 6*A1*), resulting in a sublinear increase in excitation with eTC spike number (*S_N_* = 0.54 ± 0.03 for *N* ≥ 2; *p* < 0.05 in Wilcoxon matched-pairs signed-rank test; Fig. 6*B*). Such depression occurring on a rapid time scale is most commonly caused by reductions in the number of available presynaptic vesicles (Fioravante and Regehr, 2011). As expected for such a mechanism, the degree of depression was positively correlated with the first EPSC amplitude (Fig. 6*A2*) and negatively correlated with the time difference between the first and second eTC spikes in the bursts (Fig. 6*A3*). Furthermore, we found that the AMPA receptor allosteric modulator cyclothiazide (CTZ; 50–100 µM), which slowed postsynaptic AMPA receptor desensitization (116 ± 30% increase in EPSC half-width, from 3.0 ± 0.6 to 6.8 ± 1.4 ms, *n* = 5; *p* = 0.020 in paired *t* test; Trussell et al., 1993), did not alter EPSC depression (mean decrease in *Amp_2_/Amp_1_* = 0.04 ± 0.04, *n* = 5, *p* = 0.30 in paired *t* test; Fig. 6*C*,*D*). If the EPSC depression were due to desensitization of postsynaptic AMPA receptors, CTZ should have reduced the depression. Notably, the PG cells in these recordings displayed no evidence for prolonged extrasynaptic excitatory currents (Fig. 6*E*) that were characteristic of MCs in the eTC-MC pairs (Fig. 5). Selected EPSC bursts in PG cells with more than or equal to four fast EPSCs had no late current component that could be distinguished from the fast EPSCs (mean integrated current 100–200 ms after first EPSC = –10 ± 18 pA/ms, *n* = 5).

To understand how the observed differences in the dynamics of eTC-driven excitation of MCs versus PG cells translated to the balance between the two, we computed the absolute level of excitatory current in MCs versus PG cells as a function of eTC spike number (Fig. 6*F*). While single eTC spikes elicited synaptic excitatory currents in PG cells that were ∼10-fold larger than *I_extra_*, this difference dissipated and often reversed when eTCs underwent a higher number of spikes. The striking contrast in the excitatory current dynamics occurred although the eTCs displayed similar spike frequencies in the eTC-MC versus eTC-PG cell recordings (67 ± 8 Hz for 12 eTC-MC pairs; 83 ± 13 Hz for seven eTC-PG cell pairs; *p* = 0.23, unpaired *t* test). These results indicate that the difference in non-linear properties for extrasynaptic versus synaptic excitation profoundly alters the balance between the two across varying eTC spike numbers through effects that are independent of spike frequency.

### Mechanisms underlying supralinear rise in extrasynaptic excitation (below LLD threshold)

The sublinearities in the eTC-to-PG cell transmission appeared to reflect presynaptic depression in glutamate release, but we wondered what accounted for the supralinear increase in *I_extra_*. Here, we present experimental tests for five plausible hypotheses that could explain the supralinear increase in *I_extra_*, although, as will be clear, negative results were obtained in each case.

#### Presynaptic facilitation of glutamate release

One explanation for the supralinear rise in *I_extra_* is presynaptic facilitation, wherein each later spike during a spike train produces a larger extrasynaptic current by eliciting more glutamate release than the first spike (Carter and Regehr, 2000; Clark and Cull-Candy, 2002). This possibility however could be mainly excluded based on the eTC-PG cell pair recordings already presented. Because *I_extra_* appears to be due to spillover of glutamate released at eTC-to-PG cell synapses (Fig. 1*A2*), the presynaptic depression in the EPSCs (Fig. 6*A*,*B*) also meant that the glutamate release leading to *I_extra_* was depressing, not facilitating. Indeed, to highlight the fact that supralinear increases in *I_extra_* occurred despite this depression, we recalculated the indices for supralinearity in *I_extra_* from above while normalizing for the amount of glutamate released across different numbers of eTC spikes (estimated from the EPSCs in the eTC-PG cell pairs; Fig. 5*E2*).

#### Saturation of glutamate transporters

**Figure 7.**
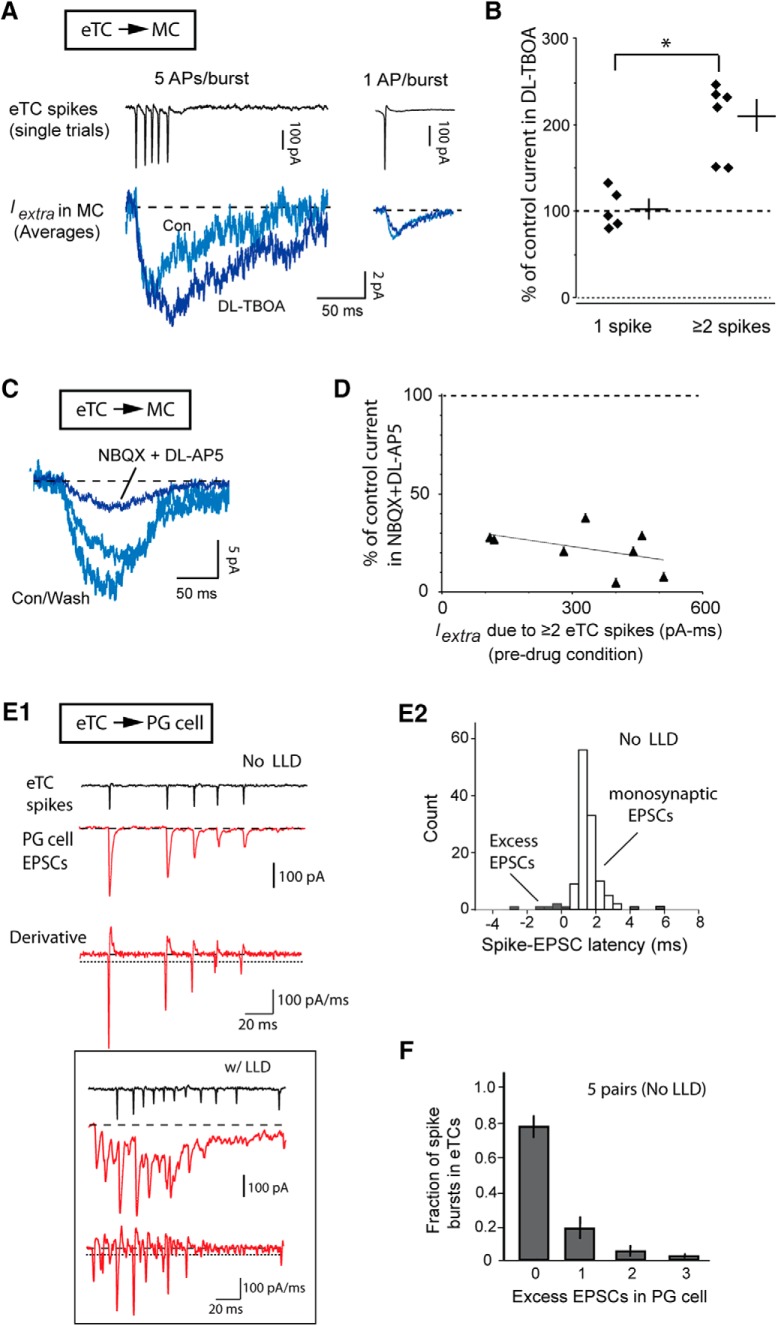
Tests of different mechanisms that could underlie the supralinear increase in extrasynaptic excitatory currents. ***A***, Example eTC-MC pair recordings showing effects of the glial glutamate transporter blocker DL-TBOA (50 µM) on *I_extra_*. Note that DL-TBOA enhanced *I_extra_* when the eTC fired five spikes (left; averages of 12–15 bursts), but not when the eTC spiked only once (right; averages of 60). An increase that is selective for the multi-spike condition is inconsistent with glutamate transporter saturation being the cause of the supralinear increase in *I_extra_* (see main text). ***B***, Summary of the effect of DL-TBOA on *I_extra_* separated by when the eTC spiked once (*n* = 5 pairs) versus two or more times (*n* = 6); *p = 0.0012 in unpaired *t* test comparing more than or equal to two- and one-spike datasets. Lines reflect mean ± SEM for the two conditions. ***C***, Example recording of *I_extra_* in MCs (averages of 12–15 trials) induced by eTC spike bursts (five spikes/burst) showing that the current was mostly blocked by the AMPA and NMDA receptor blockers, NBQX (10 µM) plus DL-AP5 (50 µM). This argued against the supralinear increase in *I_extra_* being due to recruitment of a neurotransmitter other than glutamate. ***D***, Effect of NQBX plus DL-AP5 as a function of the magnitude of *I_extra_* elicited by more than or equal to two eTC spikes. Superimposed fitted line indicates lack of correlation (*p* = 0.25), providing further evidence against the largest *I_extra_* signals being due to a non-glutamate neurotransmitter. Each of the eight data points reflects one eTC-MC pair recording. ***E***, Test for recurrent excitation and asynchronous glutamate release. ***E1***, Example analysis of a single burst event in an eTC-PG cell pair in the no-LLD condition. Note that each eTC spike had a single time-locked EPSC reflecting monosynaptic transmission, also evident in the derivative trace at bottom (horizontal dashed line = threshold). The absence of excess events argued against recurrent excitation or asynchronous glutamate release being the cause of the supralinear increase in *I_extra_*. Box demarcates a burst event in the same recording in which there was a cooccurring LLD; in this example we counted 12 excess EPSCs in the PG cell. ***E2***, Histogram reflecting latencies between eTC spikes and PG cell EPSCs in the no LLD-condition for the experiment in ***E1***. Latencies of 0.5–3.5 ms (open bars) were considered to reflect monosynaptic transmission from the test eTC onto the PG cell. ***F***, Distribution of the number of excess PG cell EPSCs per burst across five eTC-PG cell pair recordings under the no LLD-condition. Note that the large majority of burst events had no excess PG cell EPSCs.

The supralinear rise in *I_extra_* may have reflected less effective buffering of glutamate with repetitive stimuli due to saturation of glutamate transporters. To test this hypothesis, we compared the effect of the glial transport blocker DL-threo-β-benzyloxyaspartate (DL-TBOA; 10–100 µM) on *I_extra_* evoked by different numbers of eTC spikes (Fig. 7*A*,*B*). The saturation hypothesis predicted that DL-TBOA should cause a greater enhancing effect on *I_extra_* evoked by small eTC spike numbers, reflecting the greater efficacy of the transporters during the unsaturated condition, but we in fact obtained the opposite result. DL-TBOA was more effective at enhancing *I_extra_* at *higher* eTC spike numbers (119 ± 15% increase in integrated current for more than or equal to two eTC spikes, *n* = 6 pairs; 3 ± 11% increase for one eTC spike, *n* = 5 pairs; *p* = 0.0012 in unpaired *t* test comparing more than or equal to two and one spike datasets; see Discussion). Thus, transporter saturation did not appear to account for the supralinear rise in *I_extra_*.

#### Neurotransmitter(s) other than glutamate

The supralinearities in *I_extra_* may have reflected the recruitment of the release of excitatory neurotransmitters other than glutamate by eTC spike bursts (Ma et al., 2013). However, inconsistent with a major role for such a mechanism, MC currents evoked by eTC spike bursts (more than or equal to two spikes) were mainly blocked by antagonists of AMPA and NMDA receptors (NBQX, 10–20 µM, plus DL-AP5, 50–100 µM; 78 ± 4% reduction in integrated charge, *n* = 8; *p* = 0.01 in Wilcoxon matched-pairs signed-rank test; Fig. 7*C*,*D*). Also, no correlation was observed between the size of *I_extra_* and the effect of the blockers (*p* = 0.25). A role for a neurotransmitter other than glutamate in driving the supralinearities predicted that the larger *I_extra_* signals would be less sensitive to the antagonists. It is notable that there was a modest residual current in NBQX plus DL-AP5 (∼22% of the total), which suggests a possible contributing role for a non-glutamate neurotransmitter in driving *I_extra_*. However, the low magnitude of the residual current indicates that a non-glutamate neurotransmitter does not have a major role in driving *I_extra_*. Also, there are other explanations for the residual current, for example glutamate-dependent activation of metabotropic glutamate receptors (Heinbockel et al., 2004) and/or weak electrical coupling between eTCs and MCs (Gire et al., 2012).

#### Recurrent excitation

As discussed above, *I_extra_* was distinct from the glomerulus-wide LLD, but the supralinear rise in *I_extra_* may have reflected recurrent excitation among a subset of glutamatergic neurons at a glomerulus. To test this possibility, we re-analyzed burst events recorded in eTC-PG cell pairs when there was no concurrent LLD (see Materials and Methods; Fig. 7*E*). Recurrent excitation predicted that each burst of spikes in an eTC should have had both directly-linked EPSCs in the PG cell due to monosynaptic transmission, along with a significant population of excess events reflecting glutamate release from *other* activated excitatory cells. However, we found that most eTC spike bursts (77 ± 6%, *n* = 5 eTC-PG cell pairs) had zero excess events and those bursts that did generally had only one or two (Fig. 7*F*). These results suggest that recurrent excitation could not have explained the supralinear increase in *I_extra_*. A key positive control for this analysis was provided by eTC-PG cell pairs when LLD events did occur (see boxed example in Fig. 7*E1*), when 100% of eTC spike bursts had at least five excess EPSCs in the PG cell (77 spike-burst events with LLDs pooled across three pairs). The consistently large number of excess EPSCs in PG cells here argued that the low number of excess EPSCs in the no-LLD condition was not simply because PG cells were sparsely connected to excitatory neurons.

#### Asynchronous glutamate release

A last mechanism for the supralinear rise in the extrasynaptic current that we considered was asynchronous glutamate release caused by high residual calcium following a spike train (Carter and Regehr, 2000; Nahir and Jahr, 2013). Asynchronous release would be apparent in the eTC-PG cell pair recordings as a large number of excess EPSCs that followed eTC spike bursts, but such events were rarely observed (under the no-LLD condition; Fig. 7*E*,*F*). Thus, asynchronous glutamate release did not account for the supralinear increase in *I_extra_*.

If the different mechanisms that we tested did not contribute to the supralinear increase in *I_extra_*, are there other plausible mechanisms? Within the cerebellum, where extrasynaptic transmission has been studied extensively, a number of other mechanisms have been proposed to explain the fact that extrasynaptic currents only appear with stimulus trains or with synchronous stimulation of many axons (Clark and Cull-Candy, 2002; Nahir and Jahr, 2013). These include non-linear pooling of glutamate in the extrasynaptic space and/or non-linear properties of the glutamate receptors themselves (e.g., the fact that channel opening requires the binding of two glutamate molecules). Some computational support exists for non-linear pooling of glutamate at extrasynaptic sites (Pendyam et al., 2009), but there remain no experimental tests for these alternate mechanisms as causes for a supralinear rise in extrasynaptic transmission.

### Impact of non-linear dynamics on the balance between extrasynaptic excitation and inhibition within a glomerulus

In the last portion of this study, we returned to the strikingly different dynamics that were apparent when we compared extrasynaptic excitation in eTC-MC pairs versus synaptic excitation of PG cells in the eTC-PG cell pairs (Fig. 6*F*). We wondered whether such different behaviors would be observed if a more physiologic stimulus involving activation of OSN axons were used instead of spiking in a single eTC (employed during the pair-cell recordings). Also, would the different dynamics be evident if we compared extrasynaptic excitation with the local GABAergic inhibition in a glomerulus that resulted from synaptic excitation of PG cells?

**Figure 8.**
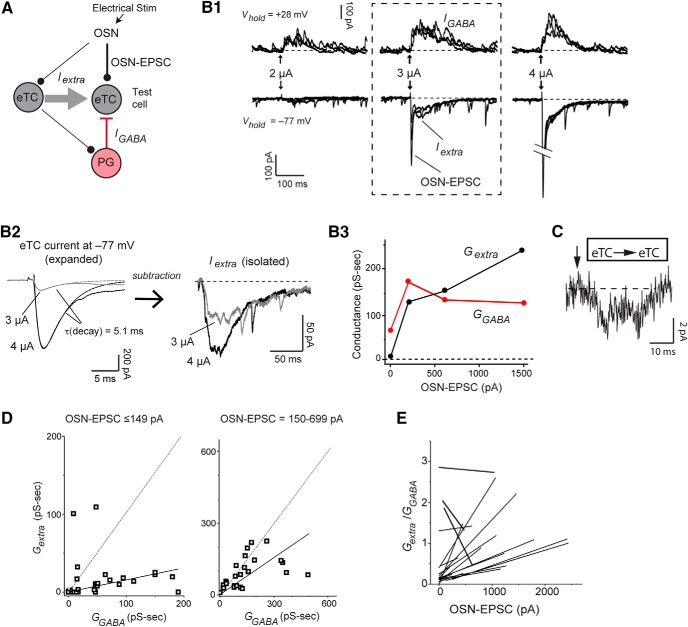
Extrasynaptic excitation and synaptic inhibition in eTCs following OSN stimulation. ***A***, Simplified circuit reflecting currents measured in a voltage-clamped eTC following stimulation of OSNs. Components include the monosynaptic OSN-EPSC, *I_extra_* due to activation of other eTCs (OSN-to-eTC-to-eTC), and a polysynaptic inhibitory current (*I_GABA_*) due mainly to feedback inhibition from PG cells (OSN-to-eTC-to-PG cell-to-eTC). Not shown is a potential feedforward pathway of inhibition mediated by ∼30% of PG cells directly excited by OSNs (Shao et al., 2009). ***B***, Example eTC current responses. ***B1***, Overlaid trials (three each) of excitatory currents (at *V_hold_* = –77 mV) and inhibitory currents (at *V_hold_* = +28 mV) evoked by OSN stimulation at three intensities (2, 3, and 4 µA). The 3-µA data in boxed region best illustrate the current components defined in ***A***. Note that at the lowest intensity (2 µA), inhibition was much larger than excitation and the OSN-EPSC was barely detectable. ***B2***, *I_extra_* (right) was isolated by fitting the rise and most of the decay of the OSN-EPSC in the composite current (left) with a sum of two exponentials, then subtracting the derived estimate of the OSN-EPSC. Average currents (five trials) at two stimulus intensities are illustrated. ***B3***, Integrated extrasynaptic (*G_extra_*) and GABAergic (*G_GABA_*) conductances as a function of OSN-EPSC amplitude for this experiment. Each point reflects mean of five trials. ***C***, Example excitatory current in an eTC evoked by single spikes in another eTC (at arrow) in an eTC-eTC pair recording (average of 12 trials). ***D***, Summary of *G_extra_* and *G_GABA_* measurements across 21 eTC recordings, sorted by amplitude of the cooccurring OSN-EPSC. Solid lines reflect linear regression fits of data points (one per recording), constrained to pass through the origin, added here for illustrative purposes. Dashed lines reflect unity. ***E***, Linear regression fits of OSN-EPSC amplitude versus *G_extra_*/*G_GABA_* ratio measurements from 19 individual eTC recordings. Each eTC recording had at least three OSN stimulation intensities sampled (actual data points not shown). Note positive slopes in all but three experiments, indicating that the conductance ratios generally rose with increasing OSN input.

To assess extrasynaptic excitation and inhibition following OSN stimulation, we opted for a strategy of recording current responses in eTCs (Fig. 8) that reside between OSNs and MCs (Fig. 1*A1*) rather than MCs themselves. eTC recordings provided a number of advantages, beginning with their anatomy. Because eTCs lack lateral dendrites, their inhibitory currents (Hayar et al., 2005) can be attributed to glomerular layer interneurons. This contrasts with MCs, which have a mixed inhibitory response, including inputs from GABAergic granule cells. Equally important was the fact that EPSCs reflecting monosynaptic transmission from OSNs (OSN-EPSCs) can be readily recorded in eTCs following electrical stimulation at a wide range of intensities (Najac et al., 2011; Gire et al., 2012; Vaaga and Westbrook, 2016). These OSN-EPSCs provided an estimate of the relative level of OSN input that could be related to the extrasynaptic excitatory and inhibitory current components isolated in the same eTC recordings (Fig. 8*A*,*B*). Constructing quantitatively meaningful OSN input-current response curves from MC recordings was much more difficult, given that OSN-EPSCs are difficult to detect or distinguish from slower current components with weak-to-moderate OSN stimuli (Najac et al., 2011; Gire et al., 2012; Vaaga and Westbrook, 2016). Importantly, eTCs, like MCs, did display clear evidence for supralinear extrasynaptic excitatory transmission from other eTCs during pair-cell recordings (single spike current = –49 ± 13 pA/ms, *S_N_* = 4.0 ± 1.0; *n* = 4 eTC-eTC pairs; Fig. 8*C*). In addition, the isolated slow component of the eTC current response to OSN stimulation was highly sensitive to DL-TBOA (183 ± 56% increase in integrated charge, *n* = 6, *p* = 0.022 in paired *t* test; data not shown), as expected for extrasynaptic transmission.

The simple prediction based on the measurements of excitatory currents in the pair-cell recordings (Fig. 6*F*) was that the eTC current response to OSN stimulation should favor inhibition at weak levels of OSN input (that presumably drive few eTC spikes), but extrasynaptic excitation should become increasingly favorable at higher levels of input. This is indeed what we found. Across 21 eTC recordings (OSN stimulation: 100 µs, 1–50 µA), weak electrical stimuli that generated small OSN-EPSCs (peak absolute amplitude ≤ 149 pA) evoked extrasynaptic excitatory and inhibitory conductances (*G_extra_* and *G_GABA_*) that were nearly always dominated by inhibition (median *G_extra_*/*G_GABA_* = 0.088, interquartile range = 0.0–0.50; *p* < 0.005 in Wilcoxon matched-pairs signed-rank test in comparison to unity; Fig. 8*B3*,*D*). In contrast, larger OSN-EPSCs were associated with the emergence of large extrasynaptic currents and conductance ratios that approached unity (median *G_extra_*/*G_GABA_* for OSN-EPSCs between 150 and 699 pA = 0.76, interquartile range = 0.38–1.31; *p* > 0.10 in Wilcoxon matched-pairs signed-rank test in comparison to unity). The shift in the conductance ratios could also be observed on an experiment-wide basis. In 19 eTC recordings in which at least three OSN input levels were sampled, values for the ratio *G_extra_*/*G_GABA_* nearly always increased as a function of the OSN-EPSC (slope of fitted lines = 3.5/nA; mean ± SE = 3.6 ± 2.0/nA; *p* < 0.01 in Wilcoxon matched-pairs signed-rank test in comparison to zero slope; Fig. 8*E*). These results suggest that the processes that led to the very different dynamics for eTC-driven extrasynaptic versus synaptic excitation in cell pairs also shape the glomerular balance between extrasynaptic excitation and synaptic inhibition in response to sensory input.

## Discussion

Recent evidence indicates that a major mechanism of sensory input-driven activation of MCs is an indirect pathway that involves intermediary eTCs that lie between OSNs and MCs (De Saint Jan et al., 2009; Najac et al., 2011; Gire et al., 2012). Moreover, signaling from eTCs to MCs appears to be extrasynaptic. Here, we have investigated eTC-to-MC extrasynaptic transmission, examining its underlying mechanisms and dynamics versus local inhibition. Our main results were: (1) extrasynaptic eTC-to-MC transmission is due to spillover of glutamate released at eTC synapses onto GABAergic PG cells; (2) extrasynaptic excitation increases supralinearly with increasing eTC spikes, differing from sublinear dynamics for synaptic excitation of PG cells due to synaptic depression; and (3) the differing dynamics of excitation alter the glomerular balance between extrasynaptic excitation and inhibition in responses to sensory input.

### Mechanisms of extrasynaptic excitation

We concluded that extrasynaptic transmission reflects spillover at eTC-to-PG cell synapses based first on patch-clamp recordings involving pair- and triple-cell combinations at the same glomerulus. In PG cell-MC pairs, we observed slow excitatory currents in MCs that were time-locked and correlated in amplitude to fast EPSCs in PG cells. The amplitude correlation was consistent with MC and PG cells responding to the same boluses of glutamate released from the eTC. Coincident MC and PG cell currents were also observed in a smaller number of pair recordings conducted in TTx. Because the mEPSCs in PG cells likely reflected glutamate release at single eTC release sites, the time-locked currents in MCs were likely due to spillover. Finally, in ultrastructural studies (Fig. 3) we observed complexes that included synapses from a labeled eTC onto likely PG cell dendrites and presumed MC dendrites in close proximity. Notably absent were astroglial processes separating the MC dendrite from the eTC-to-PG cell synapse that normally function to limit spillover of glutamate through transporters (Asztely et al., 1997; Murphy-Royal et al., 2017). It should be noted that, while our various results strongly support a mechanism for extrasynaptic transmission involving spillover at eTC-to-PG cell synapses, they do not completely exclude additional contributing mechanisms. For example, a component of the extrasynaptic signal could in principle reflect “ectopic” glutamate release sites, i.e., sites with no dedicated postsynaptic partners (Matsui and Jahr, 2003; Coggan et al., 2005).

In addition to providing evidence that eTCs can signal to MCs via spillover, our mechanistic experiments also reinforced the basic premise of this study, which was that most eTC-to-MC signaling is extrasynaptic. Prior evidence for an extrasynaptic mechanism included the absence of morphologic synapses that could support eTC-to-MC transmission (Pinching and Powell, 1971; Bourne and Schoppa, 2017). Also, in physiologic studies, eTC-to-MC excitatory currents were sensitive to a low-affinity glutamate receptor blocker (Gire et al., 2012). Here, we found in eTC-MC pair-cell recordings that a blocker of glial glutamate transporters, DL-TBOA, greatly enhanced extrasynaptic currents in MCs (*I_extra_*) when eTCs engaged in spike bursts (Fig. 7*A*,*B*). An effect of transport blockade is a signature feature of extrasynaptic transmission (Asztely et al., 1997; Isaacson, 1999; Carter and Regehr, 2000; Arnth-Jensen et al., 2002). Certainly, a caveat with interpreting the effect of DL-TBOA is that the drug may have simply recruited an extrasynaptic current not present under the original control conditions; the control current in principle could have been entirely synaptic. Nevertheless, the DL-TBOA results, when combined with prior morphologic experiments and experiments with a low-affinity glutamate receptor antagonist, make an excellent overall case that the MC current evoked by eTC spike bursts has an extrasynaptic origin.

One other interesting aspect of our experiments with DL-TBOA is that we found that the drug’s effects on MC currents were not universal: DL-TBOA did not alter the smallest *I_extra_* signals evoked by single eTC spikes. One explanation for this lack of effect is that eTC-to-PG cell synapses have few surrounding glial processes separating them from nearby MC dendrites (Fig. 3). With this geometry, local glutamate transients elicited by single eTC spikes might access a nearby population of extrasynaptic glutamate receptors unaffected by glial transporters. Notably, a very similar explanation has been suggested for extrasynaptic signaling in the cerebellum, where DL-TBOA also has its smallest effect on weak extrasynaptic currents (Clark and Cull-Candy, 2002).

Is the predominance of extrasynaptic signaling between eTCs and MCs an artifact of the brain slice preparation? In the hippocampus, it has been reported that certain brain slicing procedures can cause glial processes to retract (Bourne and Harris, 2012), which could promote such signaling. In this light, our ultrastructural results, which were based on eTC dye-fills in rat bulb slices prepared in the same manner as those used in the physiology experiments, are noteworthy since they showed that glial processes were present. In addition, other ultrastructural studies conducted using conventional whole-brain fixation methods (Chao et al., 1997; Kasowski et al., 1999) have reported subcompartments in glomeruli similar to ours that included unlabeled glutamatergic and GABAergic dendrites and few glial processes. Lastly, we found in physiologic studies that spillover was not a general phenomenon in our brain slices, not occurring at excitatory axonal synapses onto eTCs (Fig. 4).

### Dynamics of extrasynaptic transmission with spike trains

In our eTC-MC pair-cell recordings, we found profound changes in the amplitude of *I_extra_* with increasing eTC spike number. Single eTC spikes elicited very small *I_extra_* signals but *I_extra_* rose supralinearly with increasing spikes, such that the current elicited by a burst of five to seven eTC spikes was ∼29-fold larger. Qualitatively similar supralinear behavior has been observed for extrasynaptic transmission in other brain circuits, where trains of extracellular stimuli applied to axons can recruit large extrasynaptic currents not present with a single stimulus (Carter and Regehr, 2000; Clark and Cull-Candy, 2002; Nahir and Jahr, 2013). However, ours is the first study to establish quantitative relationships between spike number and extrasynaptic currents using pair-cell recordings where there is precise information about the number of cells excited and evoked action potentials. We also found, completely distinct from the supralinear rise of *I_extra_*, that *I_extra_* could trigger LLD events that reflect global recurrent excitation within a single glomerulus (Carlson et al., 2000; Fig. 5*A3*).

One issue that we attempted to address, with moderate success, was the underlying cause of the supralinear increase in *I_extra_*. Based on recordings in eTC-PG cell pairs that were conducted in parallel with the eTC-MC pairs, we obtained evidence *against* a number of mechanisms that contribute to supralinear glutamatergic signals in other systems, including presynaptic facilitation (Carter and Regehr, 2000; Clark and Cull-Candy, 2002), asynchronous glutamate release (Carter and Regehr, 2000; Nahir and Jahr, 2013), and recurrent excitation (McCormick et al., 2015; Dehghani et al., 2016). The supralinearities were also not due to saturation of glutamate transporters. Although experimental tests are lacking, we propose as an alternative that the supralinear increase in *I_extra_* reflected the local glutamate dynamics in the extrasynaptic space (Clark and Cull-Candy, 2002; Nahir and Jahr, 2013). A single spike should naturally lead to small glutamate concentrations at distant receptor sites, resulting in a small current, but this concentration could rise supralinearly as glutamate pools in the extrasynaptic space with successive spike events (Pendyam et al., 2009).

Recent studies have provided evidence that eTCs can excite MCs through the release the neuropeptide cholecystokinin (CCK; Ma et al., 2013). CCK, however, did not appear to play a major role in contributing to either the supralinear increase in the eTC-to-MC current or the eTC-to-MC current in general. We found that the eTC-to-MC current was mainly blocked by antagonists of ionotropic glutamate receptors, NBQX and DL-AP5. It is notable that a residual current of 22% remained in the presence of NBQX and DL-AP5. At least a component of this residual current likely reflected weak electrical coupling between eTCs and MCs (Gire et al., 2012), and it is possible that metabotropic glutamate receptors (Heinbockel et al., 2004) or a non-glutamate neurotransmitter such as CCK also contributed to the residual current. The important point, however, is that NBQX and DL-AP5 blocked a large majority of the eTC-to-MC current, indicating that the major underlying mechanism involved activation of ionotropic glutamate receptors.

### Balancing extrasynaptic excitation with synaptic inhibition

One of our most critical findings for understanding the function of extrasynaptic excitation came from comparing the dynamics of *I_extra_* in MCs with the cooccurring synaptic signals in PG cells that were recorded in eTC-PG cell pairs. In contrast to the supralinear increase in eTC-to-MC transmission, synaptic excitation of PG cells rose sublinearly with increasing eTC spike number due to presynaptic depression. These differing dynamics in glutamatergic excitation also had consequences for the balance between extrasynaptic excitation and synaptic inhibition within a glomerulus following OSN stimulation. In experiments in which we had precise information about the relative level of OSN input at a glomerulus (from the OSN-EPSC in an eTC), we found that inhibition dominated at low OSN input levels but strong OSN input recruited large extrasynaptic signals while inhibition saturated.

What might a changing balance between extrasynaptic excitation and synaptic inhibition in a glomerulus mean for odor coding? One intriguing hypothesis builds on a long-standing idea that the olfactory bulb functions to decorrelate signals arising from differing odors based on their differing affinities to individual odorant receptors (ORs; Yokoi et al., 1995; Mori et al., 1999). Assuming that the measures for stimulus strength that we used in our study (eTC spike number or OSN-EPSC amplitude) could be surrogates for an odor’s OR affinity, our results would imply that a low-affinity odor would mainly drive PG cell-mediated inhibition at the glomerulus coding for that OR, blocking an output, while a higher affinity odor would drive enough extrasynaptic excitation to overcome inhibition. In effect, a single glomerulus could act as a “threshold” that favors high affinity odors. Such a mechanism for decorrelating odors operating at one glomerulus could work alone or in parallel with more commonly-proposed lateral inhibitory mechanisms mediated by GABAergic granule cells (Yokoi et al., 1995; Luo and Katz, 2001; Tan et al., 2010; Koulakov and Rinberg, 2011; Yu et al., 2014).

While our study is the first to provide experimental support for a potential mechanism underlying a single-glomerulus thresholding mechanism, other supportive evidence exists. For example, in computational studies, a model of a single glomerulus was effective in decorrelating simulated odors that bound to a single OR with differing affinities (Cleland and Sethupathy, 2006; Cleland and Linster, 2012). In addition, *in vivo* studies employing optogenetic neural silencing methods pointed to a major role for uniglomerular PG cells in odor-evoked suppression of MCs/TCs and little role for granule cells (Fukunaga et al., 2014). Future studies are needed to sort out whether our proposed single-glomerulus thresholding mechanism operates as a biologically meaningful way to decorrelate natural odor stimuli or perhaps has another function such as noise rejection (Field and Rieke, 2002).

## Synthesis

Reviewing Editor: Zoltan Nusser, Institute of Experimental Medicine Decisions are customarily a result of the Reviewing Editor and the peer reviewers coming together and discussing their recommendations until a consensus is reached. When revisions are invited, a fact-based synthesis statement explaining their decision and outlining what is needed to prepare a revision will be listed below. The following reviewer(s) agreed to reveal their identity: NONE. Note: If this manuscript was transferred from JNeurosci and a decision was made to accept the manuscript without peer review, a brief statement to this effect will instead be what is listed below.

The major concerns of the reviewers were two-fold: one concerned the significance of the study and how broad the readership might be. As a general policy of eNeuro, this Journal does not reject scientific papers that employs state-of-the-art techniques and contains solid data based on the predicted impact.

The other major concern was the unknown mechanism of the supralinear increase of the excitatory current in MCs despite the short-term depression of the direct synaptic EPSCs in PG cells. The authors have made a significant effort to reveal the mechanisms during their revision. Despite their effort, they could not provide an unequivocal explanation of their data. One reviewer acknowledged this effort, but the other felt that without knowing the mechanism the impact of the paper is limited. This comes down again to the issue of perceived significance and impact. If the experiments performed were solid and if the issue is more complex than it was originally perceived, it is then acceptable that this MS does not provide a definite understanding of the mechanisms of the supralinearity.

The authors provide the following explanation of why ionotropic GluR blockers only block 78% of the current: ‘These include glutamate released from eTCs activating metabotropic glutamate receptors (Heinbockel et al., 2004), weak electrical coupling between eTCs and MCs (Gire et al., 2012), and/or a non-glutamate neurotransmitter being released directly from the eTC’. These could have been tested experimentally!

The argument that eTCs communicate to PG cells through a single release site is weak! The authors should tune this down or provide experimental evidence supporting their conclusion. That scenario is extremely rare in the CNS, therefore if the authors could provide hard evidence (see the work of A Mary et al.) that would add a significant and generally interesting statement.

Although experiments performed with uptake blockers are generally accepted as conclusive evidence for non-synaptic communication, but they are not! The fact that TBOA increases the inward current in MCs does not tell anything about the original current! How could the authors (or anyone) prove that these two currents have the same source/mechanisms of activation? The authors should write a statement in their MS that the results of TBOA experiments might not provide information about the mechanisms of the generation of the original current.

## Author Response

Below, we address the concerns raised in the prior review point by point. The comments from the review are in italics, our response in normal text. The page numbers indicated in our responses refer to the revised document with the new text highlighted in red.

The major concerns of the reviewers were two-fold: one concerned the significance of the study and how broad the readership might be. As a general policy of eNeuro, this Journal does not reject scientific papers that employs state-of-the-art techniques and contains solid data based on the predicted impact. The other major concern was the unknown mechanism of the supralinear increase of the excitatory current in MCs despite the short-term depression of the direct synaptic EPSCs in PG cells. The authors have made a significant effort to reveal the mechanisms during their revision. Despite their effort, they could not provide an unequivocal explanation of their data. One reviewer acknowledged this effort, but the other felt that without knowing the mechanism the impact of the paper is limited. This comes down again to the issue of perceived significance and impact. If the experiments performed were solid and if the issue is more complex than it was originally perceived, it is then acceptable that this MS does not provide a definite understanding of the mechanisms of the supralinearity.

As the comments suggest, we made extensive efforts to identify the mechanism(s) of the supralinear increase in the extrasynaptic eTC-to-MC current, testing five different scenarios that were based largely on studies in other systems (discussed on pp. 21-24, lines 498-575). These included: (1) presynaptic facilitation of glutamate release; (2) saturation of glutamate transporters; (3) recruitment of the release of a neurotransmitter other than glutamate; (4) recurrent excitation within the glomerular network; and (5) asynchronous glutamate release. Unfortunately, our experiments yielded negative results in each case. The studies did provide evidence against the outlined scenarios, but no positive answer was obtained.

Despite our inability to identify a mechanism to account for the supralinear increase in the extrasynaptic current, we would like to point out a number factors that we believe make our study nevertheless significant. The first is that our study makes several new and, we believe, important observations that are distinct from the question of what drives the supralinear increase in the extrasynaptic current. As outlined at the beginning of our Discussion section (p. 26, lines 629-639), our major new results included: (1) that extrasynaptic eTC-to-MC transmission is due to spillover of glutamate released at eTC synapses onto GABAergic periglomerular (PG) cells; (2) that extrasynaptic excitation increases supralinearly with increasing eTC spikes, differing from sublinear dynamics for synaptic excitation of PG cells due to synaptic depression; and (3) that the differing dynamics of excitation alter the glomerular balance between extrasynaptic excitation and inhibition in responses to sensory input. The Reviewers raised no substantial concerns with any of these conclusions. Also, as we discuss in the Discussion section, the results have implications for the understanding of both olfactory processing (p. 31, lines 747-759) as well as more “microscopic” questions around how neurons signal to each other in the nervous system (p. 29, lines 698-709).

A second point, more directly related to the issue of what causes the supralinear increase in the extrasynaptic current, is that none of the Reviewers had substantial concerns with the specific experiments conducted that tested the five potential underlying mechanisms. Thus, we believe (and apparently the Reviewers believe) that our experiments provided solid evidence against the scenarios. The Reviewers raised some concern about the experiments with glutamate receptor antagonists that tested for a role for a neurotransmitter other than glutamate (see below for more discussion), but, even with these specific experiments, no objections were raised about our fundamental conclusion: A neurotransmitter other than glutamate could not have explained a large majority of the supralinear increase in the extrasynaptic current.

A third and final point is that we propose an alternate mechanism for the supralinear increase in the extrasynaptic current (pp. 23-24, lines 565-575) that appears to be plausible based on computational studies in other systems (Pendyam et al., 2009), but this mechanism is experimentally untestable unfortunately. This is a scenario in which glutamate itself accumulates in the extrasynaptic space in a non-linear fashion with repeated action potentials. In one experimental study conducted in the cerebellum (Nahir and Jahr, 2013), the authors concluded that glutamate accumulation in the extrasynaptic space likely contributed to the supralinear increase in the extrasynaptic current that they measured (at least in some of their neurons) only after they obtained negative results in experimental tests of other mechanisms. This is essentially what we have done in our analysis of the extrasynaptic current in the olfactory bulb.

The authors provide the following explanation of why ionotropic GluR blockers only block 78% of the current: ‘These include glutamate released from eTCs activating metabotropic glutamate receptors (Heinbockel et al., 2004), weak electrical coupling between eTCs and MCs (Gire et al., 2012), and/or a non-glutamate neurotransmitter being released directly from the eTC’. These could have been tested experimentally!

The Reviewer(s) are correct that we could have tested for some of the possible mechanisms accounting for the 22% residual current. We however decided not to engage in these experiments for several reasons:

(1) Our results with ionotropic glutamate receptor blockers clearly demonstrate that the most important underlying mechanism of the eTC-to-MC current involves ionotropic glutamate receptors. None of the Reviewers appear to take issue with this conclusion, i.e., the Reviewers at no point claim that the ~78% block by the glutamate receptor blockers somehow calls into question our conclusion that most of the eTC-to-MC current must be due to a glutamatergic mechanism. Given that our study makes several other major points about the nature of the glutamatergic transmission between eTCs and MCs and between eTCs and PG cells (see above), we believe that identifying all of the mechanisms that contribute to a relatively small residual current would be beyond the scope of our study.

Certainly, another related factor contributing to our decision is the high level of difficulty of the experiments. The studies would require performing an extensive set of new pair-cell recordings that also remain intact long enough for a complete pharmacological analysis. Given the issue at hand - what explains the ~22% residual current - we believe that such an exhaustive effort is not warranted.

(2) Of the candidate mechanisms that we listed in the paper for the residual current (see Reviewer's quote above), the one that is the most likely contributor is electrical coupling between eTCs and MCs. In our own experiments, and under identical conditions, we have found that a step hyperpolarization in one cell in an eTC-MC pair elicits a small hyperpolarization in the other cell, indicative of electrical coupling (Gire et al., 2012). We in principle could have tested here whether the residual MC current evoked by depolarization of eTCs was affected by pharmacological blockers of gap junctions but such blockers are notorious for their non-specific effects on transmitter receptors and other types of ion channels. Combining this uncertainty together with the difficulty of the pair-cell recordings (see Point 1), we do not believe that tests of gap junction drugs on a small residual current are warranted.

(3) In our prior rebuttal letter to the review of the Journal of Neuroscience manuscript, we provided some evidence that the incomplete (78%) block by NBQX+DL-AP5 may have partially been because we were using a non-saturating concentration of DL-AP5. Across the 8 pair-cell recordings that yielded the 22% value for the residual current, we used either 50 µM or 100 µM DL-AP5. When we examined the subset of our experiments that employed 100 µM DL-AP5, we observed a somewhat higher level of current block (83 {plus minus} 5%, n = 5).

Notwithstanding these points, we do agree that the issue of the residual eTC-to-MC current is deserving of more discussion in our manuscript. In the revised manuscript, we have added a new paragraph to the Discussion that more fully addresses the mechanisms of the residual current (p. 30, lines 723-734).

The argument that eTCs communicate to PG cells through a single release site is weak! The authors should tune this down or provide experimental evidence supporting their conclusion. That scenario is extremely rare in the CNS, therefore if the authors could provide hard evidence (see the work of A Mary et al.) that would add a significant and generally interesting statement.

We are confused by this comment, as, at no point in our paper, do we claim that eTCs communicate to PG cells through a single release site. From the wording of the statement, it appears that the Reviewer is implying a mechanism in which each eTC excites PG cells through glutamate release at a single release site (at a single synapse).

Perhaps the Reviewer is referring to the following two sentences in the Discussion (p. 27, lines 647-650):

“Coincident MC and PG cell currents were also observed in a smaller number of pair recordings conducted in TTx. Because the mEPSCs in PG cells likely reflected glutamate release at single eTC release sites, the time-locked currents in MCs were likely due to spillover.”

Here we are claiming that it is likely that mEPSCs recorded in the presence of the sodium channel blocker tetrodotoxoin likely reflected glutamate release at a single glutamate release site from an eTC onto a PG cell. We agree that these experiments are equivocal with respect to what underlies the mEPSCs - as we did not block calcium channels in these studies - and hence we have added the qualifying term “likely”. However, we do not believe that it would be “extremely rare” that mEPSCs recorded in TTx would reflect a single release site. In addition - and most importantly - we are not using our mEPSCs recordings to claim that eTCs communicate to PG cells through a single release site.

Although experiments performed with uptake blockers are generally accepted as conclusive evidence for non-synaptic communication, but they are not! The fact that TBOA increases the inward current in MCs does not tell anything about the original current! How could the authors (or anyone) prove that these two currents have the same source/mechanisms of activation? The authors should write a statement in their MS that the results of TBOA experiments might not provide information about the mechanisms of the generation of the original current.

The Reviewer here raises an excellent point. Enhancement of an inward current by blockers of glutamate uptake such as TBOA has been widely used to argue that a current has an extrasynaptic source (Asztely et al., 1997; Isaacson, 1999; Carter and Regehr, 2000; Arnth-Jensen et al., 2002), but the presence of an enhanced current does not necessarily mean that the original current was extrasynaptic. In the revised manuscript, we have added a few sentences explaining this caveat (pp. 27-28, lines 670-675)

At the same time, we would like to emphasize that the effects of DL-TBOA are only one of three pieces of evidence that point toward eTC-to-MC signaling being due to an extrasynaptic mechanism. The other studies, from prior publications (Gire et al., 2012; Bourne and Schoppa, 2017), include morphological evidence that there are no or extremely few synaptic contacts from eTCs onto MCs and, also, the physiological effects of a low affinity glutamate receptor antagonist on the eTC-to-MC current (p. 27, lines 660-670). Thus, while there is the caveat mentioned by the Reviewer about interpreting the effect of DL-TBOA on the current, the overall body of evidence is quite solid.
